# Genetic structure of wild pea (*Pisum sativum* subsp. *elatius*) populations in the northern part of the Fertile Crescent reflects moderate cross-pollination and strong effect of geographic but not environmental distance

**DOI:** 10.1371/journal.pone.0194056

**Published:** 2018-03-26

**Authors:** Petr Smýkal, Oldřich Trněný, Jan Brus, Pavel Hanáček, Abhishek Rathore, Rani Das Roma, Vilém Pechanec, Martin Duchoslav, Debjyoti Bhattacharyya, Michalis Bariotakis, Stergios Pirintsos, Jens Berger, Cengiz Toker

**Affiliations:** 1 Department of Botany, Palacký University, Olomouc, Czech Republic; 2 Agricultural Research Ltd., Troubsko, Czech Republic; 3 Department of Geoinformatics, Palacký University, Olomouc, Czech Republic; 4 Department of Plant Biology, Mendel University, Brno, Czech Republic; 5 ICRISAT, Hyderabad, India; 6 Department of Life Science & Bioinformatics, Assam University, Silchar, India; 7 Department of Biology and Botanical Garden, University of Crete, Heraklion, Greece; 8 CSIRO Agriculture and Food, Wembley, Western Australia, Australia; 9 Department of Field Crops, Akdeniz University, Antalya, Turkey; Consiglio Nazionale delle Ricerche, ITALY

## Abstract

Knowledge of current genetic diversity and mating systems of crop wild relatives (CWR) in the Fertile Crescent is important in crop genetic improvement, because western agriculture began in the area after the cold-dry period known as Younger Dryas about 12,000 years ago and these species are also wild genepools of the world’s most important food crops. Wild pea (*Pisum sativum* subsp. *elatius*) is an important source of genetic diversity for further pea crop improvement harbouring traits useful in climate change context. The genetic structure was assessed on 187 individuals of *Pisum sativum* subsp. *elatius* from fourteen populations collected in the northern part of the Fertile Crescent using 18,397 genome wide single nucleotide polymorphism DARTseq markers. AMOVA showed that 63% of the allelic variation was distributed between populations and 19% between individuals within populations. Four populations were found to contain admixed individuals. The observed heterozygosity ranged between 0.99 to 6.26% with estimated self-pollination rate between 47 to 90%. Genetic distances of wild pea populations were correlated with geographic but not environmental (climatic) distances and support a mixed mating system with predominant self-pollination. Niche modelling with future climatic projections showed a local decline in habitats suitable for wild pea, making a strong case for further collection and *ex situ* conservation.

## Introduction

Genetic diversity of crop wild relatives (CWR) has only been rarely studied in natural populations. CWR are more diverse than domesticated crops because the latter have been forced through domestication bottlenecks. Nearly all current domestication models predict a reduction in genetic diversity in domesticated varieties compared to their wild progenitors [1]. In natural populations, micro-heterogeneity of habitats can maintain variation at small scales, while variation among environmentally diverse but locally homogenous sites can drive population differentiation and local adaptation. Genetic variation is also influenced by species demography and mating system. Moreover the environments into which domestication occurred were very different from those of modern agriculture, making it likely that certain wild adaptations which would be useful in today’s agriculture were not selected during domestication In order to widen the genetic and adaptive diversity of our crops [2], it is important to understand the genetic and adaptive diversity of the CWR themselves, sampling natural populations across their distribution. Such studies are increasingly desirable since the diversity of CWR is threatened both by habitat loss and climate change. Thus, there is an urgent need to expand CWR collections and to do so using methods that maximize genetic and environmental breadth [3,4]. Collections that span the full geographic and environmental range of the wild relative of a crop are more likely to capture a representative range of adaptations. The intra-population diversity of CWR collected in nature has been studied in cereals [5–7], but rarely in legume wild relatives in contrast to domesticated legume crops [8–12].

The mating system is part of this evolutionary and ecological background with manifold consequences for population genetics [13]. Most legume crops, such as pea (*Pisum sativum* L.) are predominantly self-pollinated [14]. Domestication has favoured this as it contributed to crop segregation from wild relatives, preventing wild-domestic hybridization with the accompanying loss of domesticated traits [15]. However, the papilionoid legume flower is well adapted for bee-mediated pollination [16], and there is always the possibility of out-crossing, albeit at low rates. Mixed mating, in which hermaphrodite plant species reproduce by both self- and cross-fertilization, poses a challenging problem for understanding genetic structure. Mixed mating complicates determining the distribution and variation of selfing among natural populations, the relationship with genetic diversity and the driving forces which shape mating patterns [13,17]. Geographic and climatic variables (mainly bioclimatic) are another part of the evolutionary and ecological background, impacting on the genetic structure of populations [18]. Despite commonly assumed decrease in genetic diversity in stressful environment, e.g. at the range periphery [19], genetic diversity may increase with fluctuating environmental conditions and in stressful environments [20] if selection favours genetic flexibility, whereas relatively more stable environments may favour higher average fitness of some few genotypes [21]. It is this genetic diversity that plant breeders are becoming increasingly interested in for further crop improvement through base broadening and trait introgression. The Fertile Crescent is the source of several of the world’s prominent crop species, including wheat, barley, flax, lentil, chickpea, and pea. Pea (*P*. *sativum* L.) belongs to the world’s oldest crops domesticated about 10,000 years ago in the Middle East and Mediterranean. These regions are also the area of *Pisum* genus origin and diversity [8,22].

In this study, we integrate genetic markers that capture divergence, and spatial genetic modelling approaches to disentangle the relative roles of geographic and climatic factors in shaping the population genetic structure of *P*. *sativum* subsp. *elatius* (M. Bieb.) Asch. & Graebn. (*Fabaceae*) represented by 187 individuals from 14 populations across northern part of the Fertile Crescent. Such analysis is important both from botanical perspective to estimate intra-population diversity and gene-flow associated with open pollination as well as practical aspects related to conservation of CWR and their potential use in breeding improvements.

We asked the following questions: 1) How inbred are the wild pea populations? 2) Is there evidence of gene-flow between populations? 3) Does isolation by distance or environment play a role in population differentiation?

## Material and methods

### Plant material

We sampled 14 populations (with 5 to 22 individuals per population) of wild pea (*P*. *sativum* subsp. *elatius*) in the region of south-eastern Turkey. We consider wild representatives of pea *P*. *sativum* subsp. *elatius* in broad sense, following the system of Maxted & Ambrose [23]. Population size varied (Table 1) from few solitary plants to several hundred plants. In most cases, the plants were either solitary with the closest neighbour within 10 meters, or distributed in patches of 2 to 5 plants. The number of sampled plants reflected population size estimated by habitat survey, accordingly we sampled about every 5^th^ -10^th^ plants per site in order to cover the entire population area (Table 1). Field harvested leaves taken from single plants were stored in silica gel until use. GPS positions were recorded (by handheld Garmin receiver) for several places at each locality.

**Table 1:** Collecting sites description.

Populations	Abbreviation	lat_dd	lon_dd	elevation	Population size estimates	Sample size	Population area estimation (m^2^)	Short habitat description
**Dagbasi**	Olm	38.0096	39.2962	705	100+	10	1000	grassland
**Eskiaygir**	Be	37.6645	37.7728	840	100+	11	1000	among rocks in pistacho gardens
**Buyukatli**	Buy	37.9882	39.1493	980	100+	12	5000	oak trees, lush green vegetation, humid
**Kokluce**	Kok	37.9148	38.9806	721	500+	14	1x106	limestone karst, oak trees
**Midyat**	Mid	37.3337	41.4844	807	200+	13	5000	oak trees, grassland
**Kilavuzlu**	Kek	37.632	36.8301	834	20	6	200	road side, slope
**Kozludere**	KM	37.6165	37.0797	1200	500+	15	5000	exposed road side, planted young cedar forest
**KahramanMaras**	KMW	37.6222	36.8325	782	500+	13	1x106	road size, edges of vegetation
**Baglica**	Bag	37.5264	40.713	845	100+	15	500	stony deposits, field edge
**Kebapci**	Keb	37.5359	40.5286	900	100+	19	1x106	dense oak trees
**Gurbuz**	Gur	37.6407	41.4283	825	200+	18	2x106	field edges, stone walls
**Hisarkaya**	His	37.6336	40.8891	730	500+	14	1x106	patches among oak trees, grassland
**Dogukent**	Xan	37.5729	39.8187	1430	13	5	4x106	extensive grassland, vulcanic rocks
**Yesilkoy**	Yesil	37.5983	40.485	900	500+	22	5000	grassland

### Description of habitat

The north-western Fertile Crescent in south-eastern Turkey is bordered by the Anti-Taurus mountains to the Mesopotamian lowland, separating it from the central Anatolian plateau. It is a region of rolling hills and a broad plateaus that extends into Syria. Eocene limestones with small spots of basalt flows are characteristic of this area. The limestone formation is dissected by erosion and represents a series of ridges (~100 m in height) separated by wadis (river valleys). Quaternary sediments consist primarily of wash and alluvial fan deposits, as well as relatively thin (1-m thick) and sporadic loamy slope deposits. In Sanliurfa, the average annual temperature is 18.1°C and average annual precipitation is 447 mm. The region belongs to warm Mediterranean climate (Csa) of Köppen climate types and to Irano-Turanian phytogeographical region. There are hot and dry summers (mean July temperature is 31.6°C; precipitation is 2 mm) and mild and comparatively humid winters (mean January temperature is 5.0°C, precipitation is 119 mm; www.globalbioclimatics.org) i.e. semi-humid steppe climate. The vegetation comprises (semi-)deciduous oak wood-pasture dominated by *Q*. *infectoria* subsp. *boissieri* or *Q*. *robur* subsp. *pedunculiflora* (K.Koch) Menitsky on neutral or alkaline soils with relatively high organic content [24]. The typical habitat was ungrazed or slightly grazed rocky limestone ground with scattered small (2-4m) oak trees *Quercus sp*. accompanied by *Pistacia terebinthus* L., *Corylus avellana* L., *Crataegus monogyna* Jacq., *Pyrus communis* L., *Acer campestre L*., *Ceratonia silique* L., *Paliurus spina-christi* Mill., *Cercis siliquastrum* L. The undergrowth indicates presence of gaps in the canopy and agro-silvopastoral land use. It consists of heliophilous plants that are also common in fields and open pastures: legumes represented by *Cicer (C*. *pinnatifidum* Jaub. & Spach, *C*. *reticulatum* L., *C*. *echinospermum* L.), *Lens culinaris* subsp. *orientalis* (Boiss.) Ponert, *Vicia* (*V*. *hybrida L*., *V*. *sericocarpa* Fenzl, *V*. *sativa* L., *V*. *noeana* Boiss., *V*. *narbonensis* L.), *Lathyrus* (*L*. *cicera* L., *L*. *sativus* L.), *Trifolium* (*T*. *campestre Schreb*., *T*. *spumosum* L., *T*. *cherleri* L., *T*. *pilulare* Boiss., *T*. *scabrum* L.), *Medicago* (*M*. *monspeliaca* (L.) Trautv., *M*. *monantha* (C.A.Mey.) Trautv., *M*. *astroites* (Fisch. & C.A.Mey.) Trautv.), *Trigonella* (*T*. *mesopotamica* Hub.-Mor., *T*. *strangulata* Boiss., *T*. *brachycarpa* (M.Bieb.) Moris.), *Coronilla scorpioides* Willd., *Securigera securidaca* (L.) Degen & Dorfl., *Astragalus hamosus* L., *Bituminaria bituminosa* (L.) C.H.Stirt. The annual grasses (*Hordeum vulgare* subsp. *spontaneum* (K.Koch) Körn., *H*. *murinum* L., *Aegilops umbellulata* Zhuk., *A*. *columnaris* Zhuk., *A*. *neglecta* Req. ex Bertol., *Triticum boeticum* Boiss., *T*. *monococcum* L., *Avena sp*., *Elymus repens* (L.) Gould, *Poa sp*., *Lolium sp*., *Bromus sp*.) are widespread. In addition there are several drought-resistant perennial grasses (*Dactylis glomerata* subsp. *hispanica* (Roth) W.D.J.Koch, *Hordeum bulbosum* L., *Poa bulbosa* L.).

### Genome wide DARTseq analysis

Genomic DNA was isolated from approximately 100 mg of dry leaf material using the Invisorb Plant Genomic DNA Isolation kit (Invisorb, Germany) and subjected to standardized DArTseq™ analysis at Diversity Arrays Technology Ltd. Canberra, Australia using proprietary methodology. DArTseq™ represents a combination of a DArT complexity reduction methods and next generation sequencing platforms [25]. DNA samples were processed in digestion/ligation reactions principally as per Kilian *et al*. [25] but replacing a single *PstI*-compatible adaptor with two adaptors. The *PstI*-compatible adapter was designed to include Illumina flowcell attachment sequence, sequencing primer sequence and barcode region. Reverse adapter contained flowcell attachment region and *MseI*-compatible sequence. Only “mixed fragments” (*PstI-MseI*) were effectively amplified in 30 rounds of PCR using the following reaction conditions: 94° C for 1 min, 30 cycles of: 94° C for 20 sec, 58° C for 30 sec, 72° C for 45 sec and final extension of 72° C for 7 min. After PCR equimolar amounts of amplification products from each sample were bulked and sequenced on Illumina Hiseq2500 run for 77 cycles. Sequences were processed using proprietary DArT analytical pipelines. Approximately 2,500,000 sequences per barcode/sample were used for marker calling using DArT PL’s proprietary SNP algorithm (DArTsoft14).

### Molecular data analyses

Genetic analysis were performed on the DArTseq SNP dataset containing 18,397 SNP (missing data < 5%, minor allele frequency, MAF > 5%). Bayesian based clustering was performed using STRUCTURE v.2.3.4 [26] testing 4 independent runs with *K* from 1 to 15, each run with a burn-in period of 50,000 iterations and 500,000 Monte Carlo Markov iterations, assuming the admixture model. The output was subsequently visualized by STRUCTURE HARVESTER v.06.92 [27] and the most likely number of clusters was inferred according to Evanno [28]. A membership coefficient q>0.8 was used to assign samples to clusters. Samples within a cluster with membership coefficients ≤0.8 were considered ‘genetically admixed’.

As STRUCTURE analysis is affected by deviations from Hardy-Weinberg equilibrium and random mating, and is thus less suitable for inbreeding species we also analysed the data by Discriminant Analysis of Principal Components (DAPC) which relies on data transformation using PCA as a prior step to Discriminant Analysis (DA). This ensures that variables submitted to DA are perfectly uncorrelated, and that their number is less than that of analysed individuals. This avoids potential bias by allowing selfing or inbreeding rates to vary between clusters [29]. DAPC analysis was performed using R package adegenet 2.0.1. The appropriate optimal number of clusters in a dataset was set to 17 according to value of Bayesian Information Criterion (BIC). Expected heterozygosity (Hexp) for polymorphic loci in each population was computed to assess intra-population genetic diversity and Hexp distribution was visualized using the standard boxplot in R.

Principal component analysis (PCA) after applying normalization technique [30] was performed as a complementary approach. To investigate the spatial pattern of genetic variability [31], spatial principal component analysis (sPCA) was done by R package *adengenet* 2.0.1. Contrary to classic PCA where eigenvalues are calculated by maximizing variance of the data, in sPCA eigenvalues are obtained by maximizing the product of variance and spatial autocorrelation (Moran’s I index)" [31].

The phylogenetic network was calculated using neighbor-net method in SplitsTree4 [32]. Analysis of molecular variance (AMOVA) were performed using R package *poppr* 2.4.1 by amova function with clone correction option [33]. Partial selfing not only creates heterozygote deficiencies, it also generates identity disequilibria i.e. correlations in heterozygosity among different loci [34]. The value g2 expresses level of Identity Disequilibrum and is computed like the covariance of heterozygosity between markers standardized by their average heterozygosity [35]. We analysed Identity Disequilibrium on extended DArTseq SNP dataset (< 70% NA; MAF > 5%) by *inbreedR 0*.*3*.*2* R package with g2_snps function [36,37]. Because of nature of g2 selfing rate estimation only populations with heterozygote SNP frequency in population more than 1% were analysed. One hundred bootstraps were used to estimate 95% confidence intervals. Selfing rate were estimated based on g2 values according David [35].

Spatial autocorrelation analysis, inter-population pairwise fixation index (Fst) and population pairwise distance matrix calculations were performed using SPAGeDi 1.5. To avoid overloading computing capacities, randomly chosen 4000 SNPs were selected from the dataset. Pairwise kinship coefficients [38] were computed for 20 distance classes which had approximately the same number of individuals. Pairwise genetic distances between populations were calculated using linearized F_ST_ value distances, e.g., F_ST_/(1 –F_ST_) as implemented in SPAGeDi.

### GIS analysis

GPS positions were taken for altogether 59 populations of *P*. *sativum* subsp. *elatius*, distributed in the broader area of south-eastern Turkey (S1 Table). Values of 19 environmental factors (see below) were extracted based on spatial localization and inserted into the geodatabase within ArcGIS for Desktop (version 10.4; http://desktop.arcgis.com/en/).

### Climatic variables

WorldClim (http://worldclim.org/) version 2.0 was used to extract minimum, mean, and maximum temperature and precipitation for 1970–2000 [39] as well as derived bioclimatic variables (S2 Table). The bioclimatic variables represent average annual values (e.g., mean annual temperature, annual precipitation) seasonality (e.g., annual range in temperature and precipitation) and extreme or limiting environmental factors (e.g., temperature of the coldest and warmest month, and precipitation of the wet and dry quarters). A quarter is a period of three months (1/4 of the year). Data was extracted in form of monthly grids bearing the respective value of the variable in ESRI grid with a spatial resolution of 30 arc-seconds (~ 1 km) in the WGS-84 (EPSG: 4326).

### Morphometric parameters of relief

Morphometric characteristics of relief reflect the character of the locality. To obtain the altitude and variables derived from elevation data, ASTER GDEM (Global Digital Elevation Model) was generated using stereo-pair images collected by the ASTER instrument onboard Terra. Transformations of coordinate systems were conducted to acquire slope, orientation and other indexes. Several indexes were calculated using Geomorphometric and Gradient Metrics Toolbox: Compound Topographic Index (Gessler *et al*. 1995; Moore *et al*. 1993) [40,41], Heat load index [42], Integrated Moisture Index [43] as estimate of soil moisture in topographically heterogeneous landscapes and Site Exposure Index [44].

### Genetic differentiation, geography and environment

The environmental data associated with each population used for genetic analyses was firstly analysed by Principal Component Analysis (PCA) to find main environmental gradients within the data-set. Before analysis, three variables were log-transformed (Bio18, 19, Slope) to normalize their distribution. Because of strong covariation among several variables, four of them were excluded from the final analysis (Bio 9, Slope, Altitude, Site exposure Index). Geographic coordinates and altitude were correlated with first two principal components after the analysis and visualised in the ordination diagram. PCA on correlation matrix was done in Canoco 5.0 [45].

To assess whether the association between genetic distance and both geographic (isolation by distance; IBD) and environmental distances (isolation by environment; IBE) exist, three matrixes were prepared and their relationships examined using the Mantel test [46]. The geographic matrix contained pairwise geographical distances while genetic distance matrix contained paired Fst values between populations. We did not use the recommended Fst/(1-Fst) [47] because preliminary analysis showed severe distortion due to several outliers. A multivariate environmental distance matrix was calculated as Euclidean distances between the populations using the same set of variables as used in the PCA. Before calculation of the environmental matrix, variables were standardized to zero mean and unit variance.

To disentangle the effect of geography and environment on genetic distance, we additionally used a partial Mantel test to calculate the partial correlation coefficients for genetic distance as a function of either geographic or environmental distance matrix while controlling for the effect of the other distance matrix. In addition, a Mantel correlogram [46] was used to identify the scales of variation using eight geographic distance classes of equal width (50 km) and seven environmental distance classes of unequal width to overcome the problem of the low number of pairs of observations in some classes and to improve the power of the tests. The significance of the normalized Mantel coefficient was calculated using a two-tailed Monte Carlo permutation test with 9999 permutations with PASSaGE v. 2.0 [48] and the statistical significance of the coefficients in Mantel correlograms was adjusted by Bonferroni correction [46].

### Climatic niche analysis

Using the GPS data for altogether 59 populations (S1 Table), the potential climatic niche was modelled using Maxent version 3.3.3k from WorldClim extracted 19 bioclimatic variables. The potential climatic niche was projected in future climatic conditions, following in the latter case the Representative Concentration Pathway (RCP) 6.0 scenario using bioclimatic data created by the Global Climate Model CCSM (Community Climate System Model) 4.0. In order to assess (1) whether selfing of the studied populations is more common in areas of low probability of occurrence in climatic niche and (2) whether selfing of the populations is more common in areas that are in high risk of becoming unsuitable due to climate change, the probabilities of occurrence of the studied populations have been estimated in the current and future projected climatic niche. For the manipulation of GIS data, as well as the creation of figures, the packages Sp [49], Raster [50] and SDMTools [51] were employed.

## Results

### Population genetic structure

DARTseq analysis performed on set of 187 individual sampled from 14 populations resulted in 40,818 SNP markers, which upon filtering for missing values (>0.05) and minor allele frequency (MAF< = 0.05) resulted in 18,397 informative SNPs used for most of further analysis (S3 Table, S1 Dataset). Of these, polymorphic loci varied from 7.5% (Baglica) to 43.5% (Yesilkoy). 10,977 of DARTseq fragments could be annotated by shortBLAST to the *Medicago truncatula* genes and showed to be evenly distributed across the chromosomes (750 to 1400 fragments per Mt chromosome represented by 1 to 20 fragments per gene). Of these 28 SNPs were located within pea chloroplast DNA (cpDNA). The AMOVA showed that 63% of the allelic variation was distributed between populations and revealed substantial geographic differentiation. The second most important contributor was the differences among individuals within populations that contributed 19% of the allelic variation. Differentiation among populations was significant, with F_ST_ values ranging from 0.15 (Yesilkoy and Kokluce, Yesilkoy-Dogukent) to 0.94 (Kebapci—Kilavuzlu, Kebapci—Baglica), indicating wide ranging genetic structure in SE Turkish pea populations, approaching free gene exchange in the first case, to almost no overlap in the second case. Genetic distances between populations increase with geographical distances (S4 Table).

**Fig 1:**
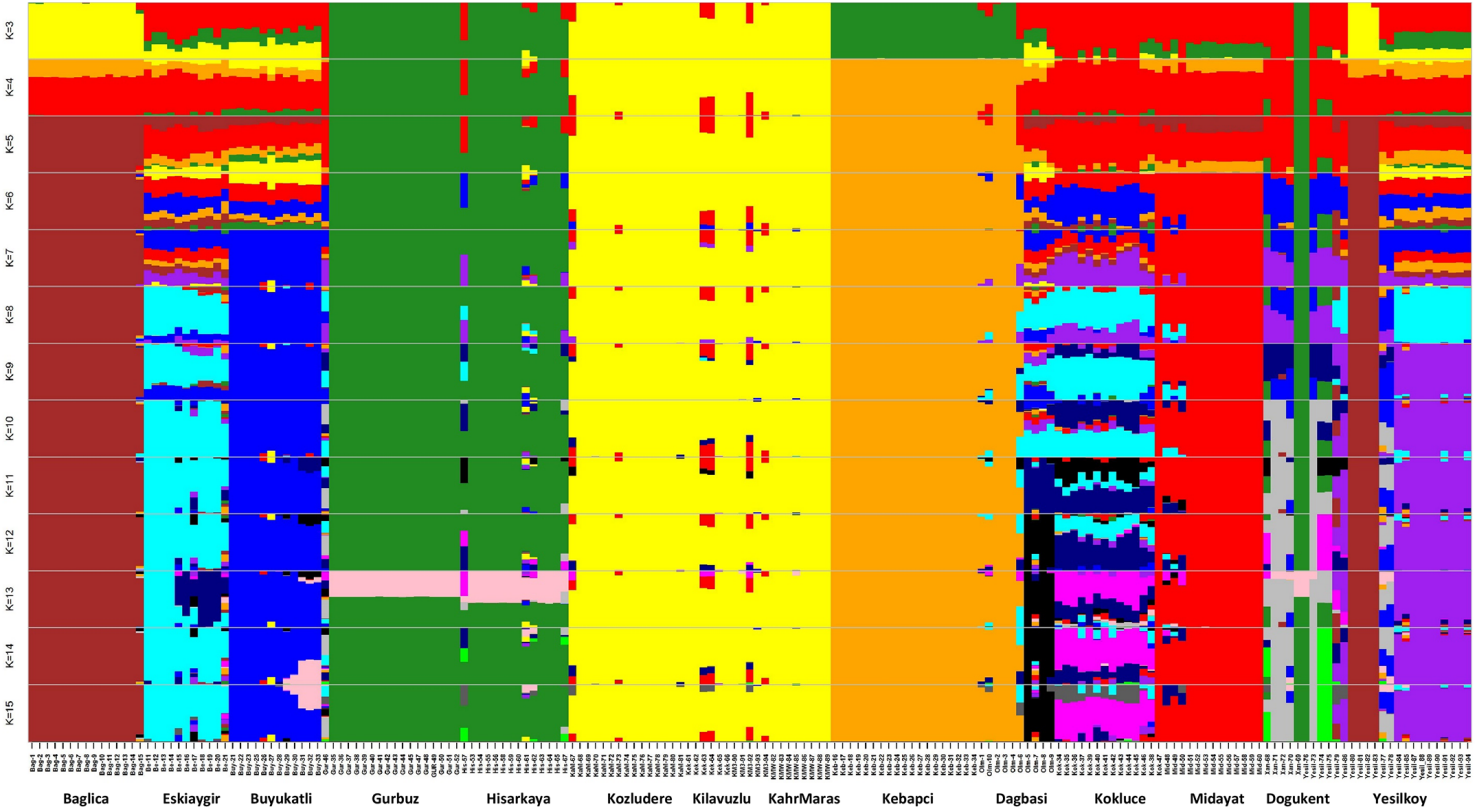
Assignment of 187 individuals to genetic clusters identified by STRUCTURE analysis, for K = 3 to 15, using 18,397 SNP.

**Fig 2:**
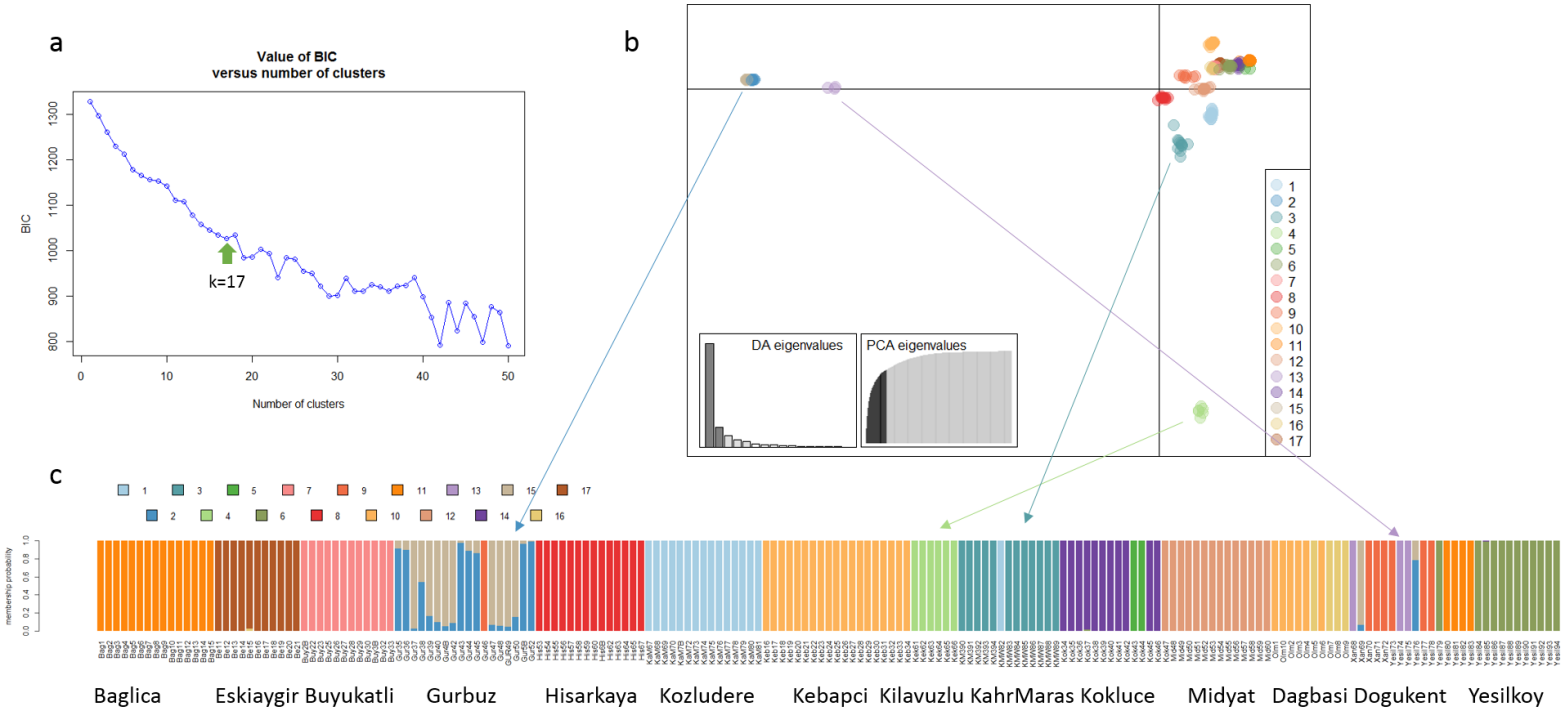
Discriminant Analysis of Principal Components (DAPC) analysis. (A) scatter plot shows genetic patterns of SNP data. The scree plots of eigenvalues (inset) indicates eigenvalues of discriminant analysis and the amount of variation contained in the different principal components (B); bar plot showing the probabilities of assignment of individuals to K = 17 genetic DAPC clusters. Arrows show clusters that are more differentiated according discriminant analysis scatter plot from other clusters and connect them with barplot.

To understand the pattern of the genetic structure, we performed a Bayesian clustering analysis in STRUCTURE and also complementary ordination analysis by Discriminant Analysis of Principal Components (DAPC). The STRUCTURE results suggested the best grouping number (K = 5) followed by 10 and 15 based on the delta K (Fig 1). At K = 5, populations of Baglica, Gurbuz-Hisarkaya, Kebapci and Kozludere-Kilavuzlu-Kahraman Maras were clearly resolved, while Eskiaygir, Dagbasi, Kebapci, Buyukatli, Dogukent and Yesilkoy contained individuals assigned to more than one cluster, indicating genetic admixture (Fig 1). At K = 10, Eskiaygir, Buyukatli, Midayat and Yesilkoy populations were further resolved. Plants from Dagbasi, Kokluce and Dogukent were physically admixed (assigned to a different cluster) at any examined K value. Individuals from these three populations were assigned both to other populations or formed separate groups indicating their genetic heterogeneity. In DAPC, which is suggested to use for self-pollinating species, allele frequency data arranged the 187 individuals into 17 clusters (Fig 2). Admixture was detected in six populations: Eskiaygir, Dagbasi, Kebapci, Gurbuz, Dogukent and particularly of Yesilkoy.

**Fig 3:**
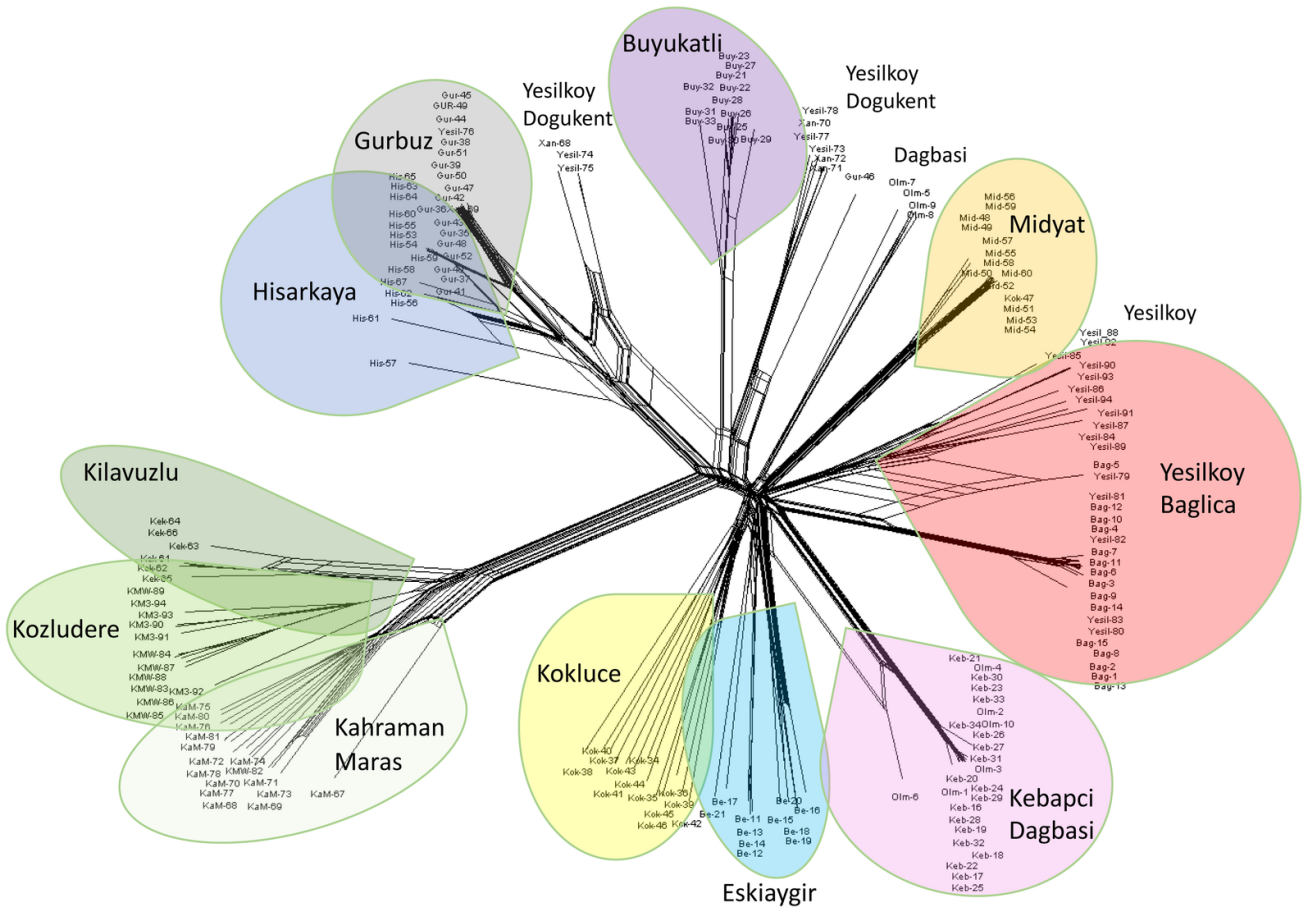
Phylogenetic network analysis calculated for DARTseq dataset containing 18,397 SNPs (NA < 5%) using neighbor-net method in SplitsTree4.

**Fig 4:**
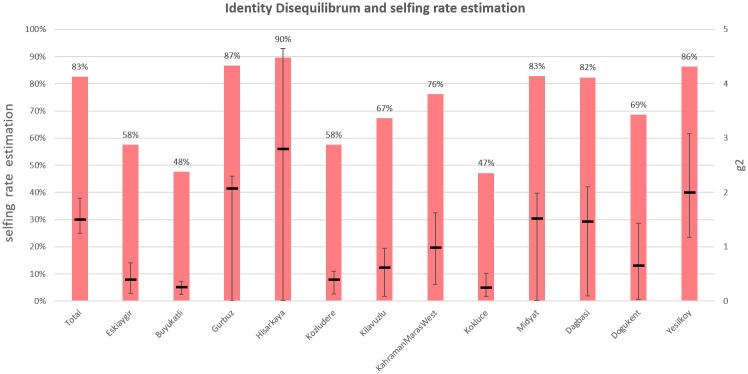
Boxplot for expected heterozygosity (*Hexp*) in population computed for polymorphic loci. Lines in boxes indicates median. Bottom and top of boxes indicate I. and III. quartiles of dataset, whiskers indicate range of data but maximally 1.5 times higher than high of box. Remaining points are outliers. The boxes are drawn with widths proportional to the square-roots of the number of polymorphic loci in the populations.

In order to further analyze the relationship among populations we conducted Principal Component Analysis of genetic data. The first two axes of PCA identified four genetic groups and explained 11% and 9.4% of the total variation, respectively (S1 Fig). Gurbuz-Hisarkaya, Kivavuzlu-Kozludere and Kahraman Maras clustered together and Kebapci with some individuals of Dagbasi population. Kokluce and particularly Yesilkoy individuals were more spread, similarly to SplitsTree (Fig 3) results. Heterozygous SNP frequency (*Hobs*) in sample ranged from 0.045 to 0.1376 in case of individual plants, and from 0.0058 (Kebapci) to 0.0356 (Kahraman Maras) as population means (Table 1, S4 Table). Moreover we assessed inter-population genetic diversity by value of expected heterozygosity (*Hexp*) in polymorphic loci. The most genetically homogenous populations was Kebapci while Dagbasi and Eskiaygir had the highest *Hexp* values. Two small sized populations, Dogukent and Kilavuzlu differed. While Kilavuzlu, had low genetic diversity, Dogukent had significantly more (Fig 4).

**Fig 5:**
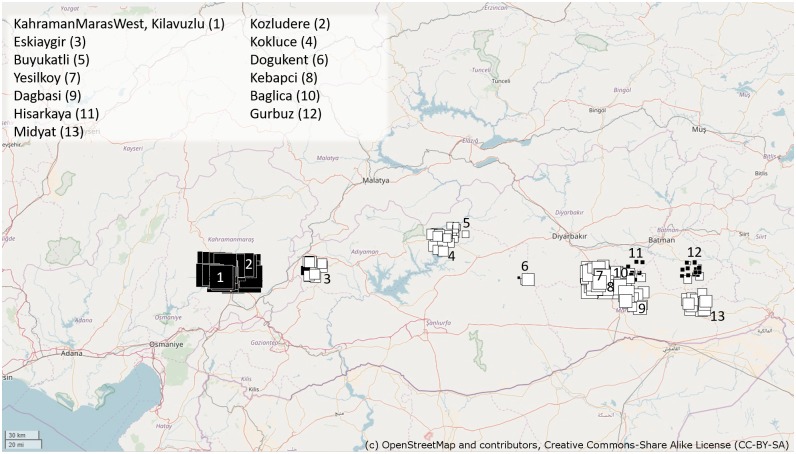
Spatial principal component analysis. Colour and size of square correlate with a score of entities in space that summarize the genetic diversity and reveal spatial structures. Positive values are represented by black squares; negative values are represented by white squares; the size of the square is proportional to the absolute value of sPC scores. Large black squares are well differentiated from large white squares, while small squares are less differentiated (Jombart *et al*. 2008). Background map is from public domain source: OpenStreetMap and contributors, available under CC-BY-SA license, downloaded at http://www.openstreetmap.org/”,.

To visualize this genetic structure in a geographic context we conducted spatial PCA (sPCA). This analysis summarized the genetic diversity and revealed spatial structures. There was a strong east-west gradient with overlap in Eskiagir, 22 km from Kilavuzlu-Kahraman Maras (Fig 5). More precisely, the first sPCA (Fig 5) separated the Kilavuzlu- Kahraman Maras populations on the west (black squares), from other more eastern populations (white squares). To examine the effect of geography on genetic structure, pairwise kinship coefficients for 20 distance classes were plotted against mean distance of the classes (S2 Fig). The steep decline of kinship coefficient is the consequence of high genetic divergence between very close populations. There is high kinship between Kozludere, Kilavuzlu and Kahraman Maras west populations, separated by 22 km, and also between Hisar and Gurbuz populations, separated by 47 km (S2 Fig). The relationship between individuals was further visualized by SplitsTree analysis (Fig 4) which clearly indicated both physical and genetic admixture (Fst = 0.397) between Yesilkoy and Baglica populations, which are 22 km apart. Similarly, Kebapci and Dagbasi populations (36 km) share genetically related individuals, and their F_ST_ is 0.361. Five out of 21 individuals of the Yesilkoy population are grouped with other (physical admixture), more distant populations (Dogukent, 59 km) and Gurbuz or Hisarkaya (83, 36 km respectively) and four out of 10 Dagbasi individuals are unrelated. Extensive genetic admixture indicating cross-pollination was identified between the geographically closest populations located within 1 km of Kahraman Maras (KaM, KMW and Kilavuzlu), followed by Hisarkaya and Gurbuz separated by 47 km (F_ST_ = 0.573). These closely related Kahraman Maras populations were genetically the most distant from the remaining pea collection (Fig 3), reflecting their location, facilitating local, but not long distance gene-flow. Physical admixture i.e. presence of individuals from one in another population was found in case of Yesilkoy population, of which 5 individuals were admixed within Baglica (22 km), 5 individuals within Dogukent (59 km), similarly 6 individuals from Dagbasi were found within Kebapci (36 km) population.

### Estimation of selfing rate

**Fig 6:**
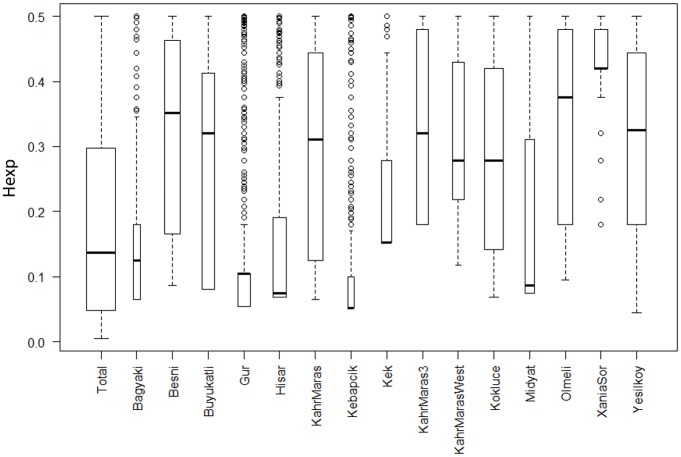
Selfing rate estimation by identity disequilibrium analysis. Black lines are value of g2 that expresses level of Identity Disequilibrium with 95% confident intervals computed using 100 bootstraps. Red bars show estimation of selfing rate based on g2 values.

As there is long standing debate about wild pea pollination systems, we estimated the selfing rate based on Identity Disequilibrium. Two populations (Kebapci and Baglica) were excluded from this analysis, as these had extremely low level of heterozygosity (S1 Table) which would influence the analysis. The remaining populations have selfing rates from 47% in Kokluce to 90% in Hisarkaya. The average selfing rate was estimated to be of 83% (Fig 6). Estimation of inbreeding coefficient by FIS was similar yet different in some samples ranging from 44% (Dogukent) till 91% (Gurbuz).

When estimated population size and area were plotted against percentage of heterozygous loci (Fig 4), weak positive relationship (R^2^ = 0.3 and 0.38, respectively) was found i.e. the larger the population, the larger the heterozygosity (with the exception of two small populations (n<20) at Kilavuzlu and Dogukent, Table 1).

### Association between genetic diversity, geographic and environmental parameters and climatic niche

**Fig 7:**
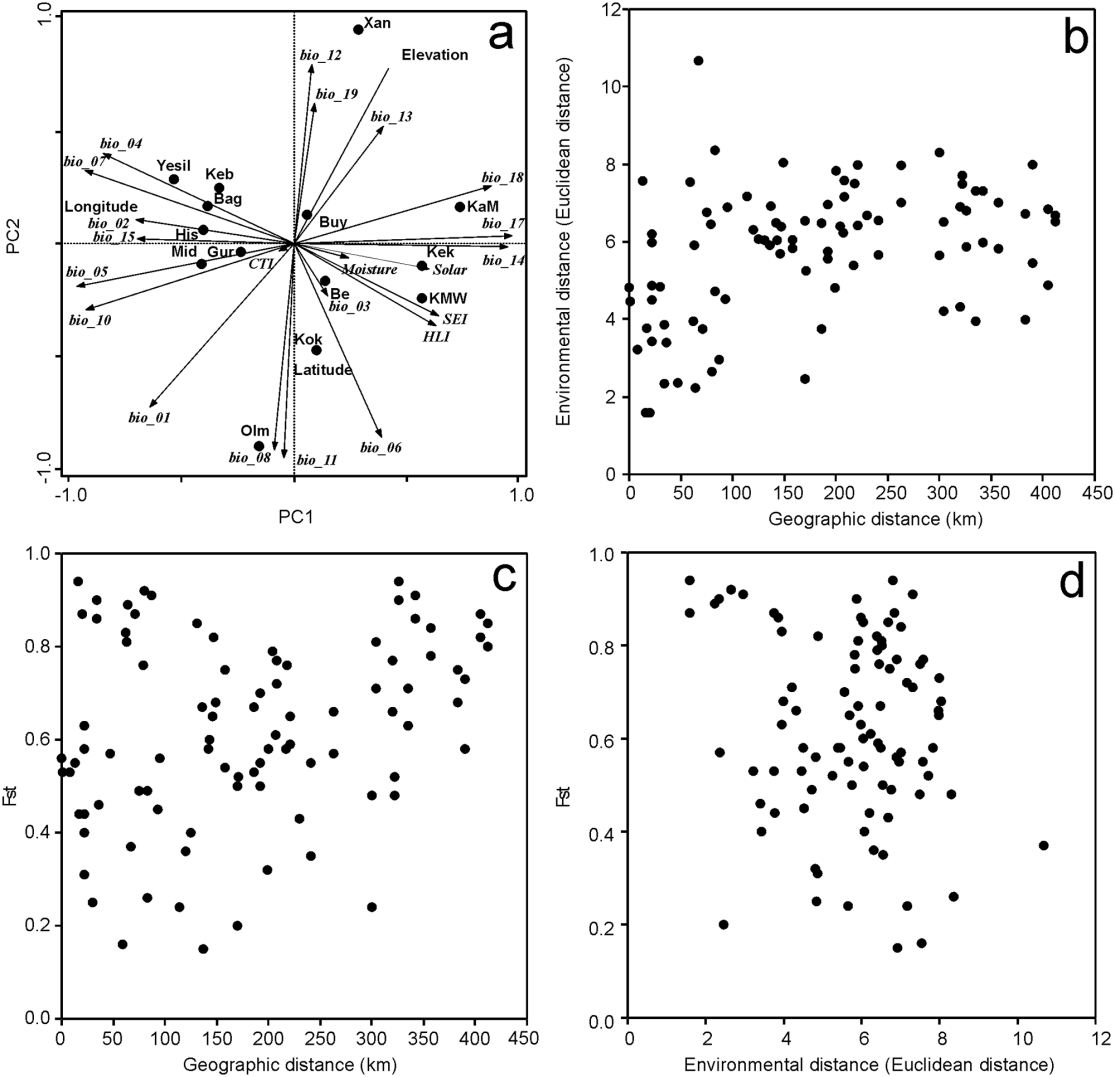
**A**) Principal component analysis of environmental data at studied sites. Geographic coordinates and elevation were correlated with the first two principal components after the analysis. First two axes explain 61% of total variation (1. axis: 39%, 2. axis: 22%). (**B**) Relationship between pairwise environmental and geographic distances. (**C**) Relationship between Fst distances and geographic and (**D**) environmental pairwise distances. Explanations: Bio_1 = Annual Mean Temperature, Bio_2 = Mean Diurnal Range (Mean of monthly (max temp—min temp)), Bio_3 = Isothermality (Bio_2/Bio_7), Bio_4 = Temperature Seasonality (standard deviation), Bio_5 = Max Temperature of Warmest Month, Bio_6 = Min Temperature of Coldest Month, BIO7 = Temperature Annual Range (Bio_5–Bio_6), Bio_88 = Mean Temperature of Wettest Quarter, Bio_9 = Mean Temperature of Driest Quarter, Bio_100 = Mean Temperature of Warmest Quarter, Bio_11 = Mean Temperature of Coldest Quarter, Bio_12 = Annual Precipitation, Bio_13 = Precipitation of Wettest Month, Bio_14 = Precipitation of Driest Month, Bio_15 = Precipitation Seasonality (Coefficient of Variation), Bio_16 = Precipitation of Wettest Quarter, Bio_17 = Precipitation of Driest Quarter, Bio_18 = Precipitation of Warmest Quarter, Bio_19 = Precipitation of Coldest Quarter, CTI = Compound Topographic Index, HLI = Heat load index, IMI = Integrated Moisture Index, SEI = Site Exposure Index. For explanations see Methods and Fick and Hijmans (2017).

The first principal component (PC1) of environmental variables was dominated by bioclimatic variables associated with east-west geographic gradient (longitude), particularly temperature and precipitation seasonality (Fig 7A). Sites on the left of the ordination are eastern locations characterised by higher temperature and precipitation seasonality and higher maximal temperature of warmest quarter and warmest month, while those on the right are higher altitude western locations with lower temperature and precipitation ranges, but with higher precipitations during warmest and driest quarters, and higher heat load index and solar radiation. PC2 separates sites by altitude, i.e. two northern, low lying sites (Dagbasi (Olm), Kokluce (Kok)) with relatively dry and warm climate in the lower part of the ordination, and Dogukent (Xan), the highest altitude site with low mean temperature and high precipitation in the upper part of the ordination diagram (Fig 7A). In summary, positions of sites in the ordination diagram roughly reflect their geographic positions and elevation.

To assess whether the geographic or the environmental difference drives the genetic divergence among populations, isolation-by distance (IBD) and isolation-by-environment (IBE) tests were conducted using the Mantel test. Genetic and geographic distance were significantly correlated (r = 0.275, P = 0.020), suggesting the IBD (Fig 7C), clearly visible at intermediate geographic distances (S3 Fig). In contrast, genetic and environmental distance were not significantly correlated (r = -0.117, P = 0.391), suggesting absence of IBE (Fig 7D) despite significant correlation between environmental and geographic distance matrices (r = 0.377, P = 0.003; Fig 7B). After controlling for confounding effects of environment, no change in IBD was found (partial Mantel test, r = 0.372, P = 0.012). Correlation between genetic and environmental distance remained non-significant after removing the confounding effect of geography (partial Mantel test, r = -0.309, P = 0.152). Significant overall Mantel test of geographic-environmental distance was caused by significant positive correlation between environmental and geographic distance at the smallest geographic scale (up to 50 km) while in other distance classes no relationships were found (Fig 7B, S3 Fig). Thus, even geographically distant and simultaneously genetically differentiated populations may not be ecologically differentiated (Fig 7B), while some environmentally rather similar sites are genetically well differentiated (Fig 7D, S3 Fig).

**Fig 8:**
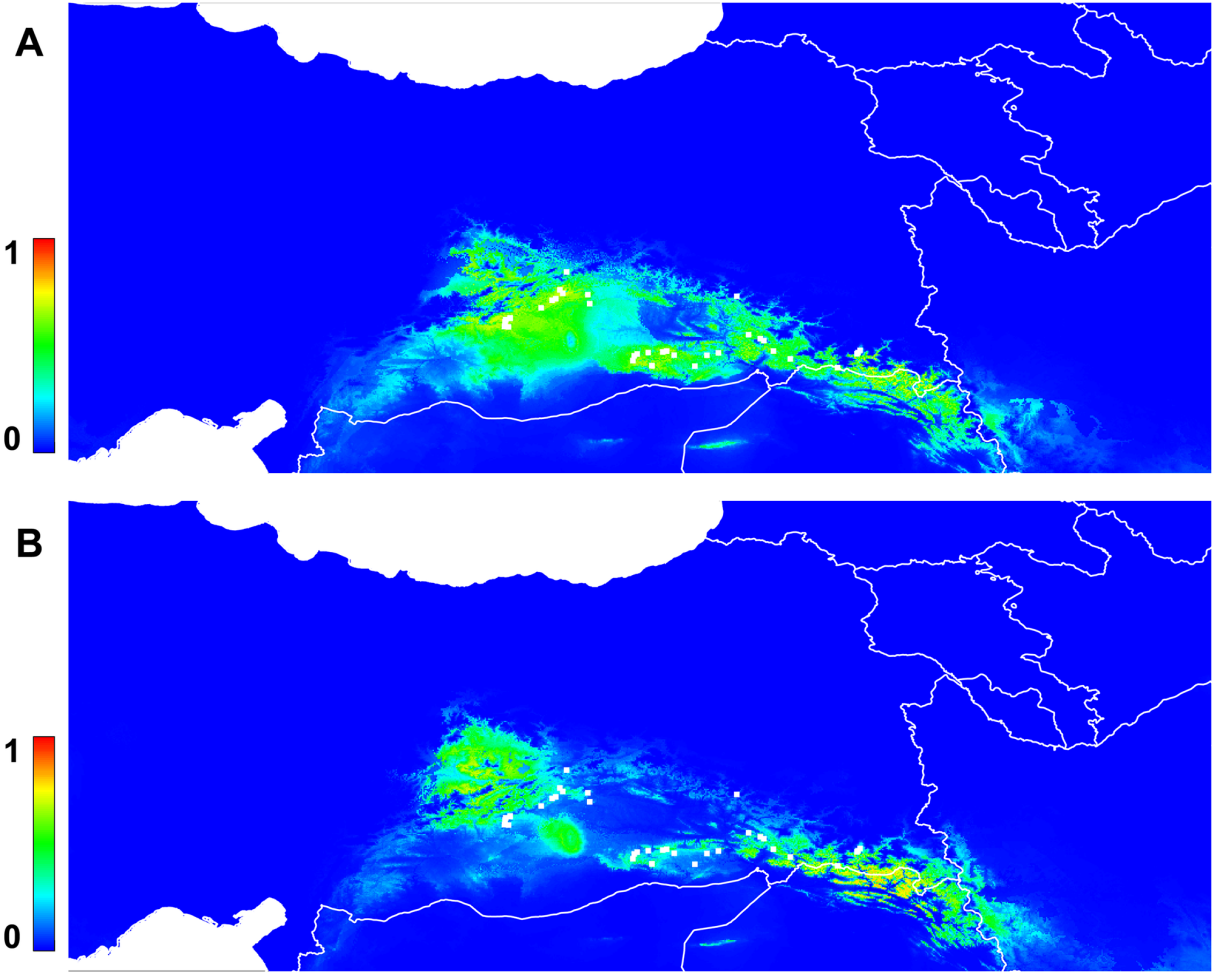
**A**) Predicted potential distribution of the populations of *P*. *sativum* subsp. *elatius* in the northern part of Fertile Crescent based on the climatic niche modelling results. Colder colours (bard blue equals 0) correspond to lower probabilities of occurrence, while warmer colours (red colour equals to 1) correspond to higher probabilities of occurrence (created with MaxEnt 3.3.3k). White squares represent the occurrence points that were used in the model. **B**) Projected potential distribution of the populations of *P*. *sativum* subsp. *elatius* in the northern part of Fertile Crescent based on the climatic niche modelling results for the year 2070. Colder colours correspond to lower probabilities of occurrence, while warmer colours correspond to higher probabilities of occurrence (created with MaxEnt 3.3.3k). White squares represent the occurrence points that were used in the model. The country borders plotting was created with R 3.2.2., the package *rworldmap*, distributed under a GPL-2 licence. Data of country borders are from Natural Earth data v 1.4.0, which are public domain.

The potential distribution of *P*. *sativum* subsp. *elatius*, as modelled (AUC = 0.780) using its recorded populations, is presented in Fig 8A. A clear shift can be observed in the projected future (Fig 8B), with areas of high potential suitability moving away from the current points of occurrence for the species, and a local decline in habitats suitable for wild pea. The mean selfing values of the studied populations do not correlate with the climate induced changes of habitat suitability for wild pea (S4 Fig).

## Discussion

While *ex situ* genetic diversity has been extensively studied in pea [20,52,53], to the best of our knowledge this is the first trial on natural populations, where the study of genetic diversity pattern of wild pea is attempted in a geographic and climatic context. While genetic variation is much larger between than within populations, the relationship between populations is clearly influenced by geographic distances. Wild pea in south-eastern Turkey has a fragmented distribution in fields, along stone walls, orchards and oak-pistachio open woodland [54]. Population size ranges from few to several hundreds of individuals, mostly separated by dozens of kilometres. It is anticipated that human activities over millennia fragmented habitats [55] and affected connectivity between populations. However in contrast to the more widespread grasses it is unlikely that wild pea formed large populations even before the intervention of humans [6]. This is partially supported by our data, where even close populations are differentiated, suggesting no present and perhaps no past connectivity. Accordingly, AMOVA analysis found the highest genetic variation (63%) between populations. Both Bayesian STRUCTURE, ordination DAPC and PCA, and distance based SplitsTree analysis detected well separated population groups (Figs 1, 2 and 3). This indicates low gene flow resulting in structured genetic diversity pattern at local scales. On other hand, physical admixture e.g. presence of individuals from one population in another (Figs 1 and 2, S1 Fig) can be explained by anthropogenic disturbance in combination with demographic population interactions. Disturbance can often drive extinction–recolonization dynamics in natural populations. Similar physical and genetic admixtures were observed in wild barley [56] explained by anthropogenic effects of human and animal mediated transport around sympatric domesticated crops. In contrast to wild pea, in wild barley most of the genetic variation is distributed within populations (67%) and less between populations (33%) [60]. Accordingly, our results in wild pea highlight the importance of sampling widely across populations in order to capture the genetic structure effectively.

Genetic differences between wild pea populations were correlated with geographic distance (Fig 7A), and F_ST_ values (S4 Table) point to barriers to gene flow. Habitat fragmentation is the most likely scenario. Moreover, there is a correlation between population size and genetic diversity [57], implying the presence of an extinction vortex, where the drop in population size lowers genetic diversity. Taken together, habitat fragmentation can lead to strong genetic drift. This is a possible scenario in our study. Wild pea was likely to be a more common species in the past that has declined due to habitat change caused by extensive deforestation, land conversion, animal overgrazing and trampling, resulting in land erosion and desertification. The human exploitation of landscape in the Middle East began over 10k years ago with the establishment of agro-pastoral communities and continues today [55, 58] affecting most species including CWR [59].

We hypothesize that several mechanisms of population stabilization are playing a role in the genetic structure of wild pea, such as the maintenance of a soil seed bank and self-fertilization. As in many Mediterranean annuals, wild legumes can form substantial soil seed banks comprising seeds with strong physical dormancy [60,61], depending on temperature and humidity patterns [62]. Seed dormancy and dispersal are adaptive traits to escape from stress in time and space, protecting populations against false breaks before the sufficient water availability [62,63], and also play a role in bet hedging against catastrophic loss within any given season. Thus seed banks can maintain genetic diversity in small populations [64] as reported in *Medicago sativa* subsp. *falcata* [65]. The capacity for selfing reduces the need for a compatible mate to maintain the species, and is particularly important in small populations with limited capacity for outcrossing. Small populations are likely to be less attractive to pollinators and may thus suffer from pollinator limitation and subsequent seed set reduction [66]. Self-pollination, as a mechanism of reproductive assurance, may compensate for the negative effects of small population size on pollinator attraction.

### Wild pea pollination and the mixed mating system

While domesticated pea is usually considered as a highly self-pollinating species [67,68], cross-pollination does occur in wild and cultivated forms [69,70, 71]. Most legumes including pea possess flowers capable of outcrossing [14]. Kosterin & Bogdanova [69, 71] demonstrated that the pea pistil remains competent after anthesis, supporting the possibility of cross-pollination. Indeed, a study of cultivated pea in Pakistan identified seven Diptera, two Hymenoptera, two Lepidoptera and one Coleoptera species as pollinators [72]. Field studies show that pea pollen may be dispersed over distances of several hundred meters [67,68]. The outcrossing rates we report in the current study (10–53%) are much higher than reported in other CWR studies (wild cowpea, 1–9.5% [10]; *Medicago truncatula*, 3–5% [9]). However some within population cross-pollination can be hidden due to high genetic uniformity allowing plants to outcross without detectable heterogeneity and heterozygozity.

Thus, self-pollination in wild pea populations is not a process, which has been favoured in domestication, but a component of the mixed mating system. This feature is valuable for breeders trying to confront the decline of pollinators. The insect-aided outcrossing allows the exploitation of heterosis potential in crops but, in the absence of pollinators, a minimum yield is achieved. This provides reproductive assurance while allowing a high level of outcrossing when pollinators are not a limiting factor [14 and references therein].

Differences in allele frequencies among wild rice populations separated by only 15 km within the same river system were found [7]. We see similar patterns in the present study. A spatial genetic structure was found for proximate wild pea populations up to maximum of 60 km, which reflects a decreased likelihood to find related individuals as distance between populations increases. The genetic relationship of some studied populations can be explained either by existing gene flow via pollen or seeds or by historical connectivity disrupted relatively recently by human activities. We propose that later scenario is more likely the case in wild pea.

Similarly to our study, a high inbreeding rate was found in self-pollinated wild rice populations [7]. Conversely in wind pollinated species forming large populations, such as wild barley, a high level of gene flow was reported over large distances [5]. Nevertheless phenotypic and genetic differentiation over small geographic scales have also been reported in Israel. The Evolution Canyon exhibits significant phenotypic and genetic differentiation between the two slopes, and suggests a strong and constant differential selection pressure to abiotic stress [19].

The heterogeneity found within populations including self-pollinated species [56] also highlights the importance of sampling strategies for germplasm collections [73] in order to capture and preserve the genetic diversity. Currently, *ex situ* held wild pea accessions originate from limited number of individuals [8], are prone to the genetic erosion [25]. In the context of climate change, individual populations might contain important adaptive traits [2].

### Genetic structure of pea is not correlated with climatic variation

The interplay between historical land use and heterogeneous environmental conditions has given rise to considerable plant biodiversity in the Mediterranean [58], and rainfall gradients place considerable selection pressure on wild populations [60]. In our study isolation-by-distance but not isolation-by-environment plays a role on genetic differentiation, suggesting that the current pea populations might be shaped by non-selective forces. Absence of IBE seems surprising at first look because the environmental gradient is related to longitude (Fig 7A) that is also major factor behind genetic differentiation of pea populations (Fig 3). Our data suggest (Fig 7, S3 Fig) that absence of IBE might be explained by interactions of several factors. Firstly, complex spatial structure of climatic variables caused rather fluctuation of environmental distances with increasing geographic distance for geographic distance classes > 100 km (Fig 7B, S3 Fig). It follows that over large spatial scale genetic distances reflect primarily geography, i.e. neutral, distance-based effects (Fig 7C). Secondly, despite overall lower environmental distance of geographically proximal sites, (< 100 km; Fig 7B), high variation in genetic and environmental distances were found between geographically close populations (Fig 7C and 7D). Such a pattern might be explained by (i) the strong variation in gene flow among close populations (as discussed above) probably mediated by various intensity of anthropogenic seed movement among currently isolated populations, and (ii) role of genetic drift and/or genetic bottlenecks where random fluctuation or sudden decline in population size in rather small-sized pea populations might results in increased genetic differentiation even among close populations. Our results are mostly comparable with Thormann et al. [56] who found IBD but not IBE (climate) explaining genetic structure of *Hordeum vulgare* subsp. *spontaneum* populations in Jordan. These authors interpreted the observed pattern by interplay among ruderal habitat preference, anthropogenic (zoochoric) movement of seeds, high self-pollination and much localized gene transfer. Most of these factors may apply to our *Pisum* data as discussed previously. However, direct analysis of the role of fine-scale abiotic and biotic variables (e.g., microclimate, disturbance regime or biotic interactions) on *Pisum* genetic structure is not possible because such variables are presently not available in public databases.

In contrast, several studies on various Mediterranean plants showed significant effect of IBE on genetic differentiation. Both environment (rainfall, temperature) and geography shaped genetic differentiation in wild barley in Israel [5] suggesting that both non-selective forces such as migration but also abiotic factors such as aridity gradient played major roles in the adaptation of wild barley. Environment but not geography influenced genetic differentiation in two *Salvia* species [74] and three of the four studied *Stipa* species in Jordan [75]. Both later mentioned authors argue that absence of IBD in presence of IDE suggest that gene flow between populations is rather limited by strong environmental variation between populations that may influence flowering phenology and consequently cause reproductive isolation between environmentally different populations irrespective of geographic distance between them [75,76]. All these studies were however conducted at similar or larger geographical scale but also in apparently more heterogeneous environment than our study.

### Influence of climate change on wild pea populations

Besides anthropogenic factors, we have to consider the current climate change as a reason for decline of this species. In our study, due to climate change, the areas of high suitability for potential future establishment of the wild pea are moving away from the current points of occurrence of the species (Fig 8A and 8B) and a local decline in habitats suitable for wild pea is predicted. One of the less recognized but very important impact of climate change is the effect on reproductive success of plants, both directly, through physiological damage and indirectly, through disruption of plant–pollinator interactions, as shown recently in faba bean [77]. It is possible that such changes took place over the evolutionary time including the period since last glacial maximum, but we cannot reach a safe conclusion with the current dataset. Nevertheless, we can conclude about climate change and plant mating pattern of wild pea.

Climatic niche modeling has extensively been used to identify potential areas of introduction and establishment of several species in different climate change scenarios, but as an approach is less reliable to predict the degree of establishment of the studied species in the new areas [78]. Climatic niche shifts has also been observed between plant species as a mechanism of establishment in new environments [79]. Environmental-induced elevation of selfing has been described to facilitate a niche shift when novel habitats are within dispersal range of core populations and this argument is also supported by the observation that in many species the expansion of their distribution in marginal habitats is associated with an increase in self-fertilization [80 and references therein]. Nevertheless, it doesn’t exclude a possible future role of selfing in climate induced changes and further studies are required.

Observed low heterozygosity and estimated selfing rate in wild pea natural populations support mixed mating system and predominant self-pollination of the species. Mating plasticity is not related with climate variability and there is no evidence of climate-enhanced selfing in natural populations of wild pea. Nevertheless, further studies are required for the role of the mixed mating system of wild pea in environmental change as well as for the use of this system in plant breeding.

### Conclusions

Here we show that in the northern Fertile Crescent, wild pea genetic variation is largely distributed between rather than within populations, and that differences between populations reflect geographic distance (IBD) rather than environmental distance (IBE). Accordingly, co-located populations are likely to be more similar than those more distant. Environment plays no role in the genetic structure we have detected. Because IBD rather than IBE is driving genetic structure in wild pea we conclude that most of the variation we detect within and between populations reflects genetic processes such as drift, founder effect and infrequent out-crossing with related individuals, rather than environmental selection pressure. Thus, if this variation is largely selectively neutral, we cannot assume that a diverse population of CWR will necessarily exhibit the wide ranging adaptive diversity required for further crop improvement. Human long term activities in the Middle East have severely fragmented the suitable habitat likely resulting in reduction of wild pea populations. The niche modelling with future climatic projections showed suitable areas decline and argue for further collecting and *ex situ* conservation. According to our analysis there is no evidence of climate-enhanced selfing in natural populations of wild pea. These are important insights because it suggests that for effective crop improvement we need more than a source of genetic diversity. We also need an understanding of what is influencing genetic structure, and how this interacts with phenotype. Only then do we have a chance of choosing the appropriate material to widen crop diversity by the introgression of adaptive traits.

## Supporting information

S1 Dataset Complete DARTseq dataset. (ZIP)

S1 Table GPS data for 59 wild pea populations. (PDF)

S2 Table WorldClim extracted bioclimatic variables and geographical distances of studied 14 populations. (PDF)

S3 Table Summary of DARTseq analysis. Percentage of observed (Hobs), expected (Hexp) and missing datapoints derived from all and polymorphic DARTseq loci per 14 studied populations are shown.
(PDF)

S4 Table Inter-population pairwise Fst (above diagonal, ANOVA approach) and geographical distances (bellow diagonal, km). (PDF)

S1 Fig Principal component analysis (PCA) of molecular data. (DOCX)

S2 Fig Results of spatial autocorrelation analysis showing mean kinship coefficient (Ritland 1996) between samples, that are divided into 20 distance groups according to pairwise geographical distance. Black points show mean distance of the distance groups.
(DOCX)

S3 Fig Mantel correlograms (Legendre & Legendre 2012) showing the scale of variation in the correlation of either environment with geography (a) and Fst with geography (b) and environment (c) using eight geographic distance classes of equal width (50 km) and seven environmental distance classes of unequal width to overcome the problem of the low number of pairs of observations in some classes and to improve the power of the tests. Positive correlation means higher environmental (a) or genetic (b, c) differentiation outside than inside the respective distance class. The significance of the normalized Mantel coefficient was calculated using a two-tailed Monte Carlo permutation test with 9999 permutations and the statistical significance of the coefficients was adjusted by Bonferroni correction. * P < 0.05, (*) 0.01 < P < 0.05 before significance correction.
(DOCX)

S4 Fig Selfing rates of the studied populations in relation to the probability of occurrence in the current and future (CCSM4 rcp6.0) projected climatic niche. (DOCX)
