# Correction: Genetic structure of wild pea (*Pisum sativum* subsp. *elatius*) populations in the northern part of the Fertile Crescent reflects moderate cross-pollination and strong effect of geographic but not environmental distance

**DOI:** 10.1371/journal.pone.0196376

**Published:** 2018-04-19

**Authors:** Petr Smýkal, Oldřich Trněný, Jan Brus, Pavel Hanáček, Abhishek Rathore, Rani Das Roma, Vilém Pechanec, Martin Duchoslav, Debjyoti Bhattacharyya, Michalis Bariotakis, Stergios Pirintsos, Jens Berger, Cengiz Toker

There is an error in the eighth sentence of the fourth paragraph in the Results. The correct sentence is as follows: The relationship between individuals was further visualized by SplitsTree analysis (Fig 3) which clearly indicated both physical and genetic admixture (Fst = 0.397) between Yesilkoy and Baglica populations, which are 22 km apart.

The following Acknowledgements are missing: Debjyoti Bhattacharyya (D.B.) acknowledges Department of Biotechnology, Ministry of Science & Technology, Government of India for the award and support of Associateship under Biotechnology Overseas Associateship program under NER scheme for the year 2013–2014.

There are errors in the caption for Fig 2. In addition, the captions for Figs 4 and 6 are incorrectly switched. Please see the correct captions and figures below.

**Fig 2:**
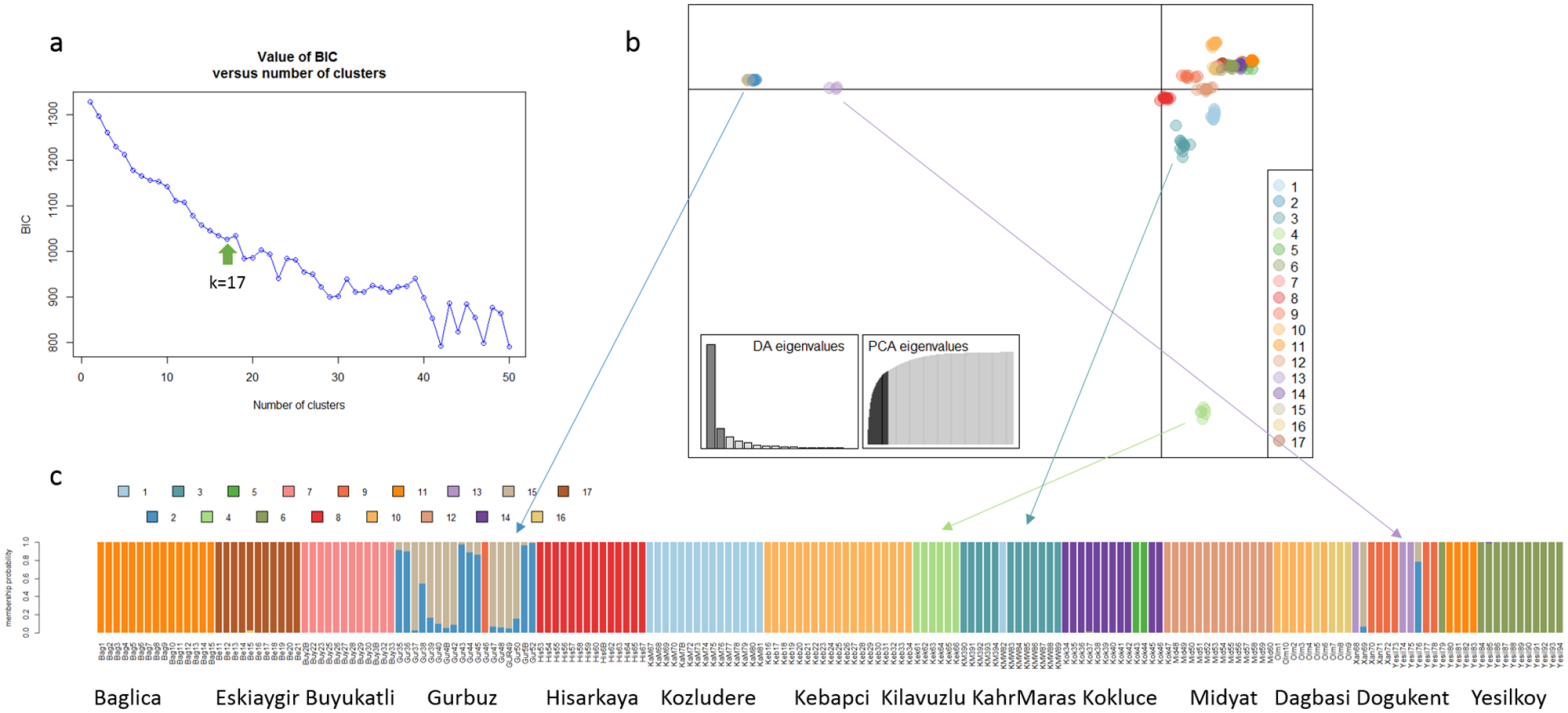
Discriminant Analysis of Principal Components (DAPC) analysis. (A) k number is selected based on BIC value for clusters up to k = 50; (B) scatter plot shows genetic patterns of SNP data. The scree plots of eigenvalues (inset) indicates eigenvalues of discriminant analysis and the amount of variation contained in the different principal components; (C) bar plot showing the probabilities of assignment of individuals to K = 17 genetic DAPC clusters. Arrows show clusters that are more differentiated according discriminant analysis scatter plot from other clusters and connect them with barplot.

**Fig 4:**
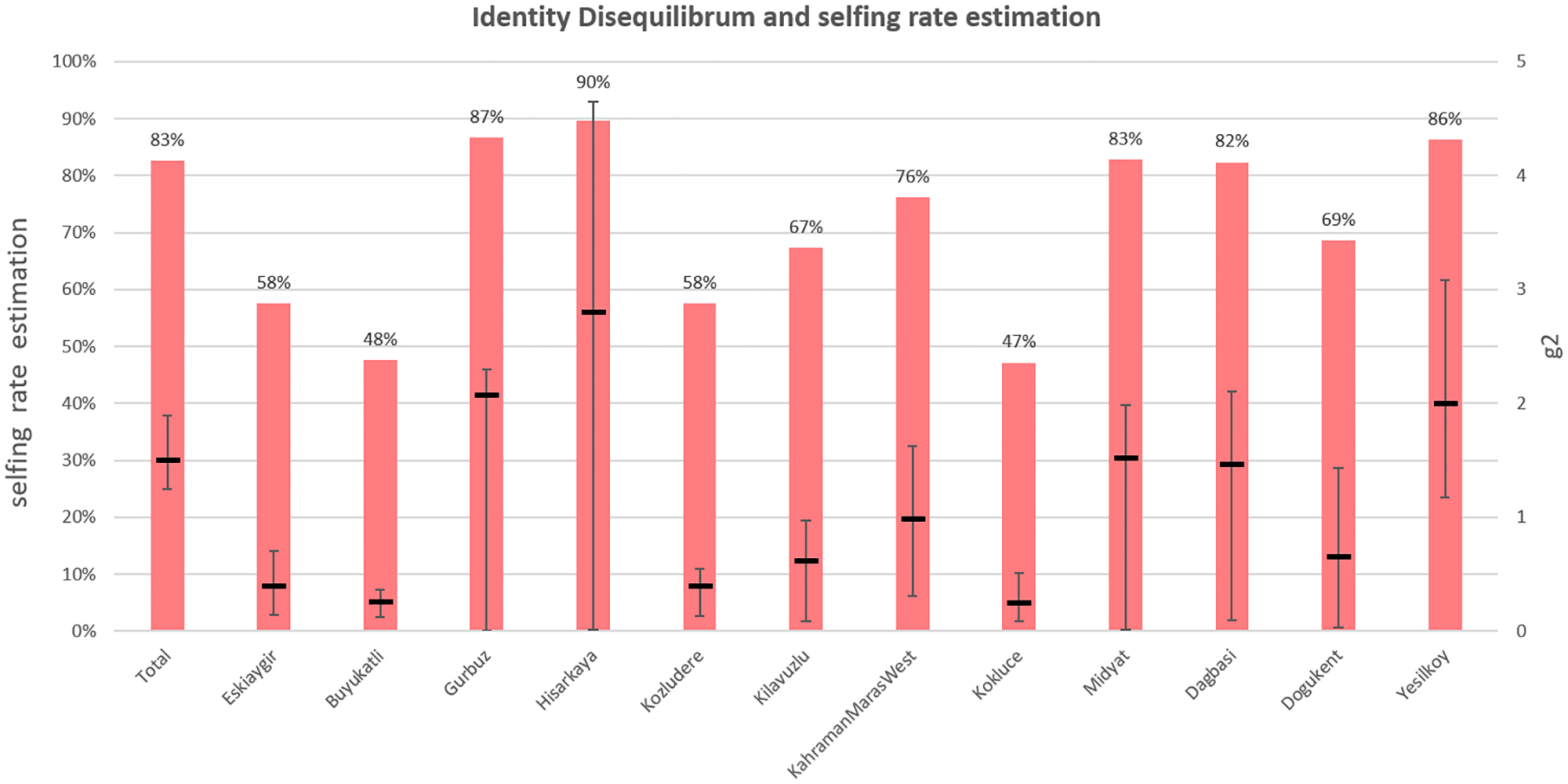
Selfing rate estimation by identity disequilibrium analysis. Black lines are value of g2 that expresses level of Identity Disequilibrium with 95% confident intervals computed using 100 bootstraps. Red bars show estimation of selfing rate based on g2 values.

**Fig 6:**
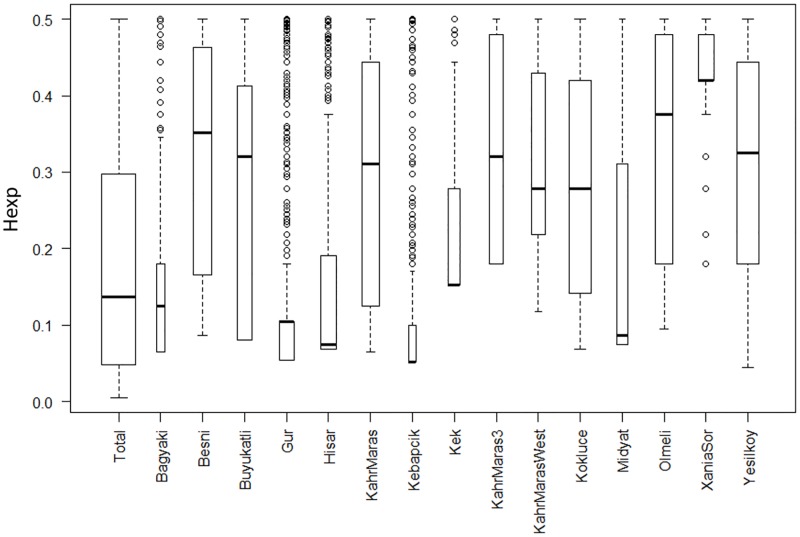
Boxplot for expected heterozygosity (Hexp) in population computed for polymorphic loci. Lines in boxes indicates median. Bottom and top of boxes indicate I. and III. quartiles of dataset, whiskers indicate range of data but maximally 1.5 times higher than high of box. Remaining points are outliers. The boxes are drawn with widths proportional to the square-roots of the number of polymorphic loci in the populations.
