# Supplementary material for: Genetic structure of wild pea (Pisum sativum subsp. elatius) populations in the northern part of the Fertile Crescent reflects moderate cross-pollination and strong effect of geographic but not environmental distance
Source: PLoS One. 2018 Mar 26;13(3):e0194056. doi: 10.1371/journal.pone.0194056 (PMC5868773; doi:10.1371/journal.pone.0194056)
Supplement: S1 Table — (PDF) [file pone.0194056.s002.pdf]

S1 Table: GPS data for 59 wild pea populations.

Nr.	Populations	lat_dd	lon_dd	elevation	
1	Dagbasi	38,00960	39,29622	705	this study - analyzed molecularly
2	Eskiaygir	37,61490	37,08429	1270	
3	Buyukatli	37,98824	39,14925	980	
4	Kokluce	37,91476	38,98063	721	
5	Midyat	37,33366	41,48435	807	
6	Kilavuzlu	37,63202	36,83008	834	
7	Kozludere	37,61652	37,07967	1200	
8	KahramanMaras West	37,62216	36,83250	782	
9	Baglica	37,52643	40,71297	845	
10	Kebapci	37,53588	40,52863	900	
11	Gurbuz	37,64067	41,42828	825	
12	Hisarkaya	37,63360	40,88912	730	
13	Dogukent	37,57287	39,81868	1430	
14	Yesilkoy	37,59833	40,48498	900	
15		37,34506	43,24265		additional populations used for niche modelling
16		37,36389	41,42576		
17		37,36724	40,87968		
18		37,37070	40,87814		
19		37,43426	40,63714		
20		37,45595	42,64077		
21		37,49636	40,65394		
22		37,50189	41,57991		
23		37,50349	41,16291		
24		37,51923	40,69223		
25		37,52129	43,48968		
26		37,53318	41,72582		
27		37,53569	43,50309		
28		37,53641	40,83262		
29		37,54456	41,01693		
30		37,55475	41,06721		
31		37,55475	41,06721		
32		37,55563	42,42590		
33		37,56468	43,52921		
34		37,56477	43,52922		
35		37,68947	42,30453		
36		37,71429	42,25953		
37		37,71438	42,25877		
38		37,71447	42,25854		
39		37,76473	42,10969		
40		37,86402	39,05476		
41		37,86913	39,00184		
42		37,89473	39,01060		
43		37,92821	39,00891		
44		37,95642	39,01755		
45		37,95647	39,01905		
46		37,95666	39,01966		
47		37,97206	39,07275		
48		37,97836	39,07547		
49		38,10712	39,46966		
50		38,15992	40,08725		
51		38,20356	39,60833		
52		38,22328	39,65478		
53		38,22339	39,65487		
54		38,25390	41,95905		
55		38,27453	40,06357		
56		38,29140	39,74428		
57		38,33601	39,70739		
58		38,33634	39,70676		
59		38,56469	39,79259		
