# Supplementary material for: Genetic structure of wild pea (Pisum sativum subsp. elatius) populations in the northern part of the Fertile Crescent reflects moderate cross-pollination and strong effect of geographic but not environmental distance
Source: PLoS One. 2018 Mar 26;13(3):e0194056. doi: 10.1371/journal.pone.0194056 (PMC5868773; doi:10.1371/journal.pone.0194056)
Supplement: S2 Table — (PDF) [file pone.0194056.s003.pdf]

S2 Table

WorldClim extracted bio_climatic variables and geographical distances of studied 14 populations

ID	1	2	3	4	5	6	7	8	9	10	11	12	13	14
Population	Bagyaki	Besni	Buyukatli	Gur	Hisar	KahrMaras	Kebapcik	Kek	KahrMarasWest	Kokluce	Midyat	Olmeli	XaniaSor	Yesilkoy
Name	Bag	Be	Buy	Gur	His	KaM	Keb	Kek	KMW	Kok	Mid	Olm	Xan	Yesil
lat_dd	37.526433	37.614904	37.988241	37.640667	37.6336	37.616517	37.535883	37.632017	37.622164	37.914759	37.333661	38.009603	37.572867	37.59833
lon_dd	40.712967	37.084293	39.149248	41.428283	40.889117	37.079667	40.528633	36.830076	36.832496	38.980631	41.484349	39.296218	39.818683	40.48498
elevation	845	1269,868042	979,932	825	730	1200	900	834	782	720,677063	807,040771	705,267639	1430	900
prec_01	102	89	78	101	100	96	103	106	110	77	99	77	112	99
prec_02	99	73	74	103	97	80	98	92	95	70	98	69	106	95
prec_03	96	75	80	103	96	87	97	88	89	76	97	72	107	95
prec_04	89	60	72	100	87	69	89	65	66	65	94	55	100	86
prec_05	43	41	53	51	44	47	45	42	42	45	46	37	63	45
prec_06	7	10	11	8	8	13	8	13	13	10	6	7	12	8
prec_07	1	2	3	1	1	4	2	4	4	2	1	1	3	1
prec_08	1	2	2	1	1	4	1	4	4	2	1	2	2	1
prec_09	3	5	4	4	3	8	3	8	7	4	3	3	5	3
prec_10	38	39	46	38	36	45	38	45	46	41	35	34	50	37
prec_11	67	63	65	75	68	70	69	72	74	59	67	52	88	69
prec_12	99	97	85	103	97	102	100	116	120	85	101	85	120	98
tavg_01	1,600000024	2,099999905	0,699999988	2,200000048	1,899999976	1,299999952	1,299999952	3,200000048	3,900000095	2,799999952	2,599999905	5,300000191	-1,100000024	1,399999976
tavg_02	4,099999905	3,700000048	2,599999905	4,300000191	4,199999809	2	3,799999952	4,199999809	5	4,5	4,5	6,5	0,300000012	3,900000095
tavg_03	8,600000381	7,900000095	7,199999809	8,800000191	8,899999619	5,900000095	8,300000191	7,699999809	8,300000191	8,899999619	9,300000191	10,19999981	5,699999809	8,399999619
tavg_04	13,89999962	13,60000038	13	14,19999981	14,30000019	11,30000019	13,60000038	13	13,5	14,19999981	14,5	15,19999981	11,19999981	13,80000019
tavg_05	19,20000076	19	18,39999962	19,39999962	19,39999962	15,89999962	18,89999962	18,10000038	18,5	19,39999962	19,60000038	20,60000038	16,70000076	19,10000038
tavg_06	25,20000076	24,20000076	24,29999924	25,29999924	26,20000076	20,70000076	25,10000038	22,89999962	23,39999962	25,29999924	26,39999962	26,29999924	22,10000038	25,5
tavg_07	30	28,20000076	28,70000076	30,39999962	30,89999962	23,20000076	29,89999962	25,29999924	25,70000076	29,70000076	30,89999962	29,89999962	26,5	30,29999924
tavg_08	29,5	27,89999962	28,20000076	29,89999962	30,10000038	23,60000038	29,29999924	26	26,60000038	29,20000076	30,29999924	29,20000076	26,20000076	29,5
tavg_09	24,39999962	24	24	25,20000076	25,10000038	21,10000038	24,39999962	23,39999962	23,89999962	25	25,60000038	25,10000038	22	24,60000038
tavg_10	17,39999962	17,20000076	16,79999924	17,89999962	17,60000038	15,19999981	17,29999924	17	17,70000076	18	18	19	15	17,29999924
tavg_11	9,100000381	9,399999619	8,699999809	10	9,699999809	8,699999809	9	10,39999962	11,10000038	10,69999981	10,5	12,30000019	7	9,100000381
tavg_12	3,5	4,5	3,599999905	4,199999809	4,099999905	3,799999952	3,299999952	5,5	6,199999809	6,099999905	5	7,199999809	1,5	3,400000095
tmin_01	-6,5	-4,599999905	-5,800000191	-5,5	-5,800000191	-5,699999809	-6,800000191	-2,900000095	-2,299999952	-3,799999952	-5,400000095	-2,599999905	-8,199999809	-6,5

ID	1	2	3	4	5	6	7	8	9	10	11	12	13	14
Population	Bagyaki	Besni	Buyukatli	Gur	Hisar	KahrMaras	Kebapcik	Kek	KahrMarasWest	Kokluce	Midyat	Olmeli	XaniaSor	Yesilkoy
tmin_02	-4	-2,900000095	-3,900000095	-3,400000095	-3,5	-4,900000095	-4,300000191	-1,899999976	-1,200000048	-2	-3,5	-1,399999976	-6,699999809	-4
tmin_03	0,600000024	1,200000048	0,699999988	1,100000024	1,100000024	-1	0,300000012	1,600000024	2,099999905	2,299999952	1,299999952	2,299999952	-1,299999952	0,5
tmin_04	5,800000191	6,900000095	6,5	6,5	6,599999905	4,400000095	5,599999905	6,900000095	7,300000191	7,699999809	6,5	7,400000095	4,199999809	5,900000095
tmin_05	11,10000038	12,30000019	11,89999962	11,69999981	11,60000038	9	10,89999962	11,89999962	12,39999962	12,80000019	11,69999981	12,69999981	9,600000381	11,19999981
tmin_06	17,20000076	17,5	17,79999924	17,60000038	18,5	13,69999981	17,10000038	16,79999924	17,20000076	18,70000076	18,39999962	18,39999962	15	17,60000038
tmin_07	24,20000076	23,10000038	24,70000076	24,89999962	25,70000076	17,79999924	24	20,29999924	20,60000038	25,5	25,10000038	24,29999924	21,39999962	24,70000076
tmin_08	23,70000076	22,89999962	24,20000076	24,39999962	24,89999962	18,20000076	23,39999962	21,10000038	21,39999962	25	24,39999962	23,70000076	21,10000038	24
tmin_09	18,60000038	19	20	19,79999924	19,89999962	15,69999981	18,5	18,39999962	18,79999924	20,79999924	19,70000076	19,60000038	16,89999962	19,10000038
tmin_10	11,60000038	12,10000038	12,80000019	12,39999962	12,39999962	9,800000191	11,39999962	12,10000038	12,60000038	13,80000019	12,19999981	13,5	9,899999619	11,80000019
tmin_11	3,299999952	4,300000191	4,699999809	4,5	4,5	3,299999952	3,099999905	5,400000095	5,900000095	6,5	4,599999905	6,800000191	1,899999976	3,599999905
tmin_12	-2,299999952	-0,5	-0,5	-1,200000048	-1,200000048	-1,700000048	-2,599999905	0,600000024	1	1,899999976	-0,800000012	1,700000048	-3,599999905	-2,099999905
tmax_01	9,600000381	8,800000191	7,199999809	9,899999619	9,699999809	8,199999809	9,300000191	9,300000191	10	9,300000191	10,5	13,10000038	5,900000095	9,300000191
tmax_02	12,10000038	10,39999962	9,100000381	12	12	8,899999619	11,80000019	10,39999962	11,19999981	11,10000038	12,39999962	14,39999962	7,400000095	11,80000019
tmax_03	16,70000076	14,5	13,69999981	16,5	16,70000076	12,89999962	16,39999962	13,80000019	14,39999962	15,39999962	17,20000076	18,10000038	12,80000019	16,29999924
tmax_04	22	20,29999924	19,5	21,89999962	22,10000038	18,29999924	21,60000038	19,10000038	19,60000038	20,79999924	22,39999962	23,10000038	18,29999924	21,70000076
tmax_05	27,29999924	25,70000076	24,89999962	27,10000038	27,20000076	22,79999924	27	24,20000076	24,70000076	26	27,60000038	28,39999962	23,70000076	27
tmax_06	33,29999924	30,89999962	30,79999924	33	34	27,60000038	33,09999847	29	29,5	31,79999924	34,29999924	34,09999847	29,10000038	33,40000153
tmax_07	35,79999924	33,20000076	32,79999924	35,79999924	36,09999847	28,70000076	35,70000076	30,20000076	30,89999962	33,90000153	36,79999924	35,40000153	31,60000038	35,79999924
tmax_08	35,29999924	32,90000153	32,29999924	35,29999924	35,29999924	29,10000038	35,20000076	31	31,70000076	33,40000153	36,09999847	34,70000076	31,20000076	35,09999847
tmax_09	30,20000076	29	28,10000038	30,60000038	30,39999962	26,60000038	30,29999924	28,29999924	29,10000038	29,20000076	31,39999962	30,60000038	27	30,10000038
tmax_10	23,29999924	22,20000076	20,89999962	23,29999924	22,79999924	20,70000076	23,10000038	22	22,89999962	22,20000076	23,79999924	24,5	20,10000038	22,79999924
tmax_11	14,89999962	14,39999962	12,69999981	15,39999962	14,89999962	14,19999981	14,80000019	15,39999962	16,20000076	14,89999962	16,29999924	17,79999924	12,10000038	14,60000038
tmax_12	9,300000191	9,5	7,599999905	9,600000381	9,300000191	9,199999809	9,199999809	10,5	11,30000019	10,30000019	10,89999962	12,80000019	6,599999905	9
vapr_01	0,540000021	0,5	0,469999999	0,529999971	0,550000012	0,430000007	0,519999981	0,5	0,519999981	0,540000021	0,540000021	0,600000024	0,400000006	0,529999971
vapr_02	0,560000002	0,540000021	0,5	0,550000012	0,569999993	0,479999989	0,540000021	0,550000012	0,569999993	0,569999993	0,560000002	0,620000005	0,419999987	0,550000012
vapr_03	0,670000017	0,660000026	0,610000014	0,670000017	0,689999998	0,589999974	0,660000026	0,670000017	0,689999998	0,689999998	0,680000007	0,75	0,519999981	0,670000017
vapr_04	0,910000026	0,850000024	0,819999993	0,899999976	0,939999998	0,75	0,879999995	0,860000014	0,889999986	0,910000026	0,889999986	0,980000019	0,699999988	0,899999976
vapr_05	1,049999952	1,039999962	1	1,039999962	1,090000033	0,939999998	1,039999962	1,080000043	1,110000014	1,100000024	1,00999999	1,159999967	0,850000024	1,059999943
vapr_06	1,080000043	1,139999986	1,080000043	1,039999962	1,110000014	1,070000052	1,080000043	1,24000001	1,289999962	1,169999957	0,980000019	1,230000019	0,930000007	1,100000024
vapr_07	1,179999948	1,269999981	1,169999957	1,120000005	1,200000048	1,220000029	1,169999957	1,440000057	1,49000001	1,269999981	1,049999952	1,340000033	1,019999981	1,190000057
vapr_08	1,149999976	1,279999971	1,159999967	1,080000043	1,169999957	1,210000038	1,149999976	1,429999948	1,49000001	1,279999971	1,029999971	1,370000005	1,00999999	1,169999957

ID	1	2	3	4	5	6	7	8	9	10	11	12	13	14
**Population**	**Bagyaki**	**Besni**	**Buyukatli**	**Gur**	**Hisar**	**KahrMaras**	**Kebapcik**	**Kek**	**KahrMarasWest**	**Kokluce**	**Midyat**	**Olmeli**	**XaniaSor**	**Yesilkoy**
**vapr_09**	0,99000001	1,080000043	0,980000019	0,939999998	1,00999999	1	0,980000019	1,169999957	1,220000029	1,090000033	0,899999976	1,169999957	0,839999974	1
**vapr_10**	0,910000026	0,930000007	0,879999995	0,889999986	0,939999998	0,850000024	0,889999986	0,959999979	0,99000001	0,970000029	0,870000005	1,029999971	0,75	0,910000026
**vapr_11**	0,75	0,74000001	0,699999988	0,74000001	0,769999981	0,660000026	0,730000019	0,74000001	0,75999999	0,779999971	0,74000001	0,839999974	0,600000024	0,75
**vapr_12**	0,620000005	0,600000024	0,550000012	0,620000005	0,639999986	0,529999971	0,600000024	0,600000024	0,620000005	0,620000005	0,649999976	0,680000007	0,469999999	0,600000024
**srad_01**	8009	7713	7726	8067	7815	7506	7972	8027	7963	7482	8201	7936	7878	7870
**srad_02**	10780	10240	10474	10708	10665	10090	10847	10486	10494	10260	10797	10765	10863	10687
**srad_03**	14710	14616	14493	14667	14580	14325	14690	14700	14705	14416	14877	14730	14727	14632
**srad_04**	18557	19616	19212	18317	18573	19175	18693	19675	19694	19265	18383	19602	18939	18734
**srad_05**	23451	23621	23412	23285	23261	23048	23592	23468	23443	23535	23415	23898	23510	23457
**srad_06**	27753	28156	27964	27700	27440	27872	27854	28050	28104	27885	27688	28146	27856	27775
**srad_07**	27735	29181	28811	28228	27768	29192	27932	29065	29052	28931	27898	28856	28445	27849
**srad_08**	25295	26340	26230	25732	25425	26324	25507	26316	26336	26223	25416	26196	25835	25423
**srad_09**	20814	21297	21108	20843	20819	21233	20964	21281	21304	21030	20817	21154	21312	20888
**srad_10**	14668	14782	14532	14741	14675	14632	14780	14849	14821	14572	14719	14815	14808	14759
**srad_11**	10175	9879	9747	10157	9936	9805	10162	10029	10014	9661	10298	9993	10030	9965
**srad_12**	7380	7047	7113	7429	7248	6823	7454	7265	7273	6865	7547	7258	7325	7291
**bio__01**	15,54583341	15,12916679	14,69583321	15,96666649	16,04999991	12,74166665	15,33749995	14,72916664	15,30416675	16,14583347	16,41249978	17,22500005	12,7500001	15,52916672
**bio__02**	13,87499955	11,70833343	10,54166641	13,1333333	12,9833332	12,38333333	13,90833344	11,07500018	11,30833348	10,75833357	13,79166627	13,38333339	12,13333355	13,42499983
**bio__03**	32,80141797	30,97442652	27,31001702	31,79983908	30,98647641	35,58429097	32,7254897	32,66961695	33,25980364	28,53669271	32,68167415	35,21929708	30,48576255	31,73758883
**bio__04**	1026,085804	959,9348118	1018,364234	1018,955186	1042,193913	837,319421	1031,318937	849,8541497	844,2626147	968,0098292	1023,872149	902,4701003	1008,090008	1038,704302
**bio__05**	35,79999924	33,20000076	32,79999924	35,79999924	36,09999847	29,10000038	35,70000076	31	31,70000076	33,90000153	36,79999924	35,40000153	31,60000038	35,79999924
**bio__06**	-6,5	-4,599999905	-5,800000191	-5,5	-5,800000191	-5,699999809	-6,800000191	-2,900000095	-2,299999952	-3,799999952	-5,400000095	-2,599999905	-8,199999809	-6,5
**bio__07**	42,29999924	37,80000067	38,59999943	41,29999924	41,89999866	34,80000019	42,50000095	33,9000001	34,00000072	37,70000148	42,19999933	38,00000143	39,80000019	42,29999924
**bio__08**	3,0333335	3,449999968	2,283333302	5,099999925	3,416666627	2,333333214	2,766666651	4,333333294	5	4,466666798	4,016666522	6,333333393	0,233333429	2,916666746
**bio__09**	27,96666686	26,68333371	27,01666673	28,46666622	28,71666622	22,68333356	27,85000006	24,88333321	25,41666667	27,96666718	28,91666635	28,05000051	24,86666679	28,13333321
**bio__10**	28,25	26,75000032	27,09999975	28,49999968	29,08333302	22,68333356	28,08333333	24,88333321	25,41666667	28,05000051	29,18333276	28,4333334	24,90000025	28,4333334
**bio__11**	3,0333335	3,449999968	2,283333302	3,566666643	3,416666627	2,333333214	2,766666651	4,333333294	5	4,466666798	4,016666522	6,333333393	0,233333429	2,916666746
**bio__12**	645	556	573	688	638	625	653	655	670	536	648	494	768	637
**bio__13**	102	97	85	103	100	102	103	116	120	85	101	85	120	99
**bio__14**	1	2	2	1	1	4	1	4	4	2	1	1	2	1
**bio__15**	78,49487607	73,81092898	68,61628356	77,24610695	77,9562504	70,02017827	77,55184918	74,00191387	75,02601983	70,57097438	78,94115203	75,265809	73,15527636	77,38313428

**bio__16**	300	259	237	307	294	278	301	314	325	232	298	231	338	292
**bio__17**	5	9	9	6	5	16	6	16	15	8	5	6	10	5
**bio__18**	9	14	16	10	10	16	11	16	15	14	8	10	17	10
**bio__19**	300	259	237	307	294	278	301	314	325	232	298	231	338	292
**Elevation ASTERGDEM 10**	849	832	977	815	743	1240	892	842	811	720	800	535	1413	838
**Aspect 10**	329,8264771	160,7099609	48,17982864	145,9540558	320,8067322	176,8201752	349,3803406	292,9887085	221,5317688	178,7811279	180	198,4349518	129,4724579	20,05609512
**Slope 10**	11,36871243	9,721237183	11,64690018	10,23233509	18,72837448	16,25156021	7,49769783	16,16245079	20,70679092	10,75999546	0,46321699	8,721133232	10,09572124	28,46773911
**Mean Slope 10**	10,09398937	8,387169838	10,12278748	10,48383808	14,32238293	16,2035656	8,234630585	20,80785751	20,54620934	8,576181412	3,350106239	8,516691208	10,29863071	27,93859863
**CTI 10km**	6,223686695	7,076970577	6,891995907	6,331533432	6,394373894	7,238569736	9,537841797	12,4289608	7,671862125	6,973255634	14,7844429	6,494283199	6,34526062	9,123461723
**HLI 10km**	0,814489007	0,839115798	0,753992796	0,83278048	0,830978513	0,869731963	0,800361276	0,857437551	0,876120687	0,848785877	0,808681846	0,841569841	0,821055889	0,703825414
**Int Moisture Index 10km**	95,00955963	85,8547821	89,86434937	85,02391052	98,873909	84,55000305	98,93087006	346,4782715	90,92131042	86,3547821	162,6260986	88,09562683	85,01912689	111,6900253
**Site Exposure Index 10km**	-9,830270767	9,175331116	-7,768682957	8,477957726	-14,51860905	16,22653198	-7,369807243	-6,316045761	15,49956989	10,75756073	0,46321699	8,273478508	6,417031288	-26,74491119
**Solar 10**	1264889,342	1467875,564	1310157,36	1451778,629	1156516,118	1579939,606	1311518,472	1248463,198	1466412,168	1466154,044	1386509,143	1417792,972	1540357,972	926020,3645

bio__1 = Annual Mean Temperature

bio_2 = Mean Diurnal Range (Mean of monthly (max temp - min temp))

bio_3 = Isothermality (bio_2/bio_7) (* 100)

bio_4 = Temperature Seasonality (standard deviation *100)

bio_5 = Max Temperature of Warmest Month

bio_6 = Min Temperature of Coldest Month

bio_7 = Temperature Annual Range (bio_5-bio_6)

bio_8 = Mean Temperature of Wettest Quarter

bio_9 = Mean Temperature of Driest Quarter

bio_10 = Mean Temperature of Warmest Quarter

bio_11 = Mean Temperature of Coldest Quarter

bio_12 = Annual Precipitation

bio_13 = Precipitation of Wettest Month

bio_14 = Precipitation of Driest Month

bio_15 = Precipitation Seasonality (Coefficient of Variation)

bio_16 = Precipitation of Wettest Quarter

bio_17 = Precipitation of Driest Quarter

bio_18 = Precipitation of Warmest Quarter

bio_19 = Precipitation of Coldest Quarter

tmin = minimum temperature (°C)

tmax = maximum temperature (°C)

tavg = average temperature (°C)

prec = precipitation (mm)

srad = solar radiation (kJ m-2 day-1)

vapr = water vapor pressure (kPa)

number behind the abbrevation = number of month e.g. prec01 = mean precipitation in January

lat_dd = latitude (°)

lon_dd = longitude (°)

Elevation ASTER GDEM = elevation from ASTER Global Dfigital Elevation Model

CTI = Compound Topographic Index (Gessler et al., 1995; Moore et al., 1993)

HLI = Heat load index (McCune & Keon 2002)

Int Moisture Index = Integrated Moisture Index (Iverson et al., 1997)

Solar = solar radiation (watt-hour per square metre (sum for 2016))

Slope = calculated slope from DEM (°)

Aspect = calculated aspect from DEM (°)

10 or 10km = diameter of buffer
