# Supplementary material for: Genetic structure of wild pea (Pisum sativum subsp. elatius) populations in the northern part of the Fertile Crescent reflects moderate cross-pollination and strong effect of geographic but not environmental distance
Source: PLoS One. 2018 Mar 26;13(3):e0194056. doi: 10.1371/journal.pone.0194056 (PMC5868773; doi:10.1371/journal.pone.0194056)

S3 Table

Summary of DARTseq analysis. Percentage of observed (Hobs), expected (Hexp) and missing datapoints derived from all and polymorphic DARTseq loci per 14 studied populations are shown

	Total (187)	Baglica (15)	Eskiaygir (11)	Buyukatli (12)	Gurbuz (18)	Hisarkaya (14)	Kozludere (15)	Kebapci (19)	Kilavuzlu (6)	Kahraman-Maras West (13)	Kokluce (14)	Midyat (13)	Dagbasi (10)	Dogukent (5)	Yesilkoy (22)
**mean Hobs**	0.03	0.01	0.02	0.02	0.02	0.02	0.04	0.01	0.03	0.02	0.07	0.02	0.03	0.07	0.03
**mean Hexp of polymorphic loci**	0.18	0.13	0.32	0.27	0.10	0.14	0.28	0.08	0.22	0.26	0.27	0.18	0.32	0.40	0.31
**missing data**	0.15	0.12	0.13	0.15	0.16	0.16	0.18	0.13	0.18	0.21	0.12	0.17	0.11	0.12	0.14
**polymorphic loci**	0.50	0.07	0.28	0.17	0.20	0.20	0.26	0.04	0.12	0.26	0.46	0.12	0.27	0.22	0.44
**samples in population**	187	15	11	12	18	14	15	19	6	13	14	13	10	5	22
**number of polymorphic loci**	40818	2962	11516	7034	8039	8271	10684	1525	4840	10629	18658	4698	11103	8811	17788

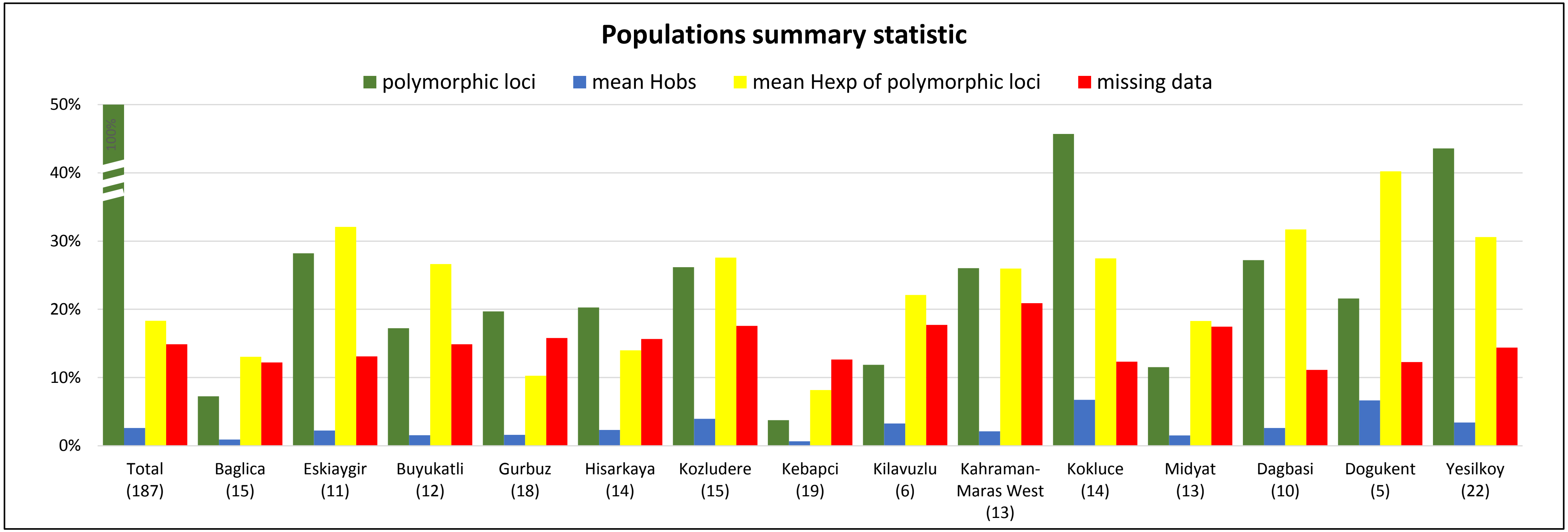

Supplement: S3 Table — Percentage of observed (Hobs), expected (Hexp) and missing datapoints derived from all and polymorphic DARTseq loci per 14 studied populations are shown. (PDF) [file pone.0194056.s004.pdf]
