# Supplementary material for: Genetic structure of wild pea (Pisum sativum subsp. elatius) populations in the northern part of the Fertile Crescent reflects moderate cross-pollination and strong effect of geographic but not environmental distance
Source: PLoS One. 2018 Mar 26;13(3):e0194056. doi: 10.1371/journal.pone.0194056 (PMC5868773; doi:10.1371/journal.pone.0194056)
Supplement: S4 Table — (PDF) [file pone.0194056.s005.pdf]

S4 Table

Inter-population pairwise Fst (above diagonal, ANOVA approach) and geographical distances (bellow diagonal, km)

	Baglica	Eskiaygir	Buyukatli	Gurbuz	Hisarkaya	Kozludere	Kebapci	Kilavuzlu	KahramanMaras West	Kokluce	Midyat	Dagbasi	Dogukent	Yesilkoy
Baglica		0.66	0.82	0.89	0.87	0.77	0.94	0.91	0.86	0.54	0.87	0.67	0.76	0.40
Eskiaygir	259		0.53	0.68	0.63	0.56	0.71	0.63	0.58	0.20	0.58	0.32	0.35	0.24
Buyukatli	147	126		0.79	0.75	0.67	0.85	0.77	0.72	0.44	0.76	0.55	0.49	0.40
Gurbuz	64	322	204		0.57	0.75	0.92	0.87	0.82	0.58	0.86	0.70	0.58	0.49
Hisarkaya	20	274	158	47		0.71	0.90	0.84	0.78	0.52	0.83	0.65	0.56	0.46
Kozludere	320	61	186	383	335		0.81	0.44	0.31	0.50	0.73	0.58	0.55	0.48
Kebapci	16	243	131	80	34	304		0.94	0.90	0.60	0.91	0.36	0.81	0.53
Kilavuzlu	342	83	208	405	357	22	326		0.53	0.55	0.85	0.65	0.66	0.52
KahramanMarasWest	342	83	208	405	357	22	326	1		0.50	0.80	0.59	0.57	0.48
Kokluce	158	110	17	217	171	170	143	192	192		0.43	0.25	0.26	0.15
Midyat	71	329	218	34	62	390	87	412	412	230		0.61	0.68	0.45
Dagbasi	23	276	167	57	29	336	36	358	358	178	54		0.37	0.24
Dogukent	79	180	75	142	95	241	63	263	263	83	149	96		0.16
Yesilkoy	22	239	125	83	36	300	8	322	322	137	93	43	59	
