# Supplementary figures and images for: Genetic structure of wild pea (Pisum sativum subsp. elatius) populations in the northern part of the Fertile Crescent reflects moderate cross-pollination and strong effect of geographic but not environmental distance
Source: PLoS One. 2018 Mar 26;13(3):e0194056. doi: 10.1371/journal.pone.0194056 (PMC5868773; doi:10.1371/journal.pone.0194056)

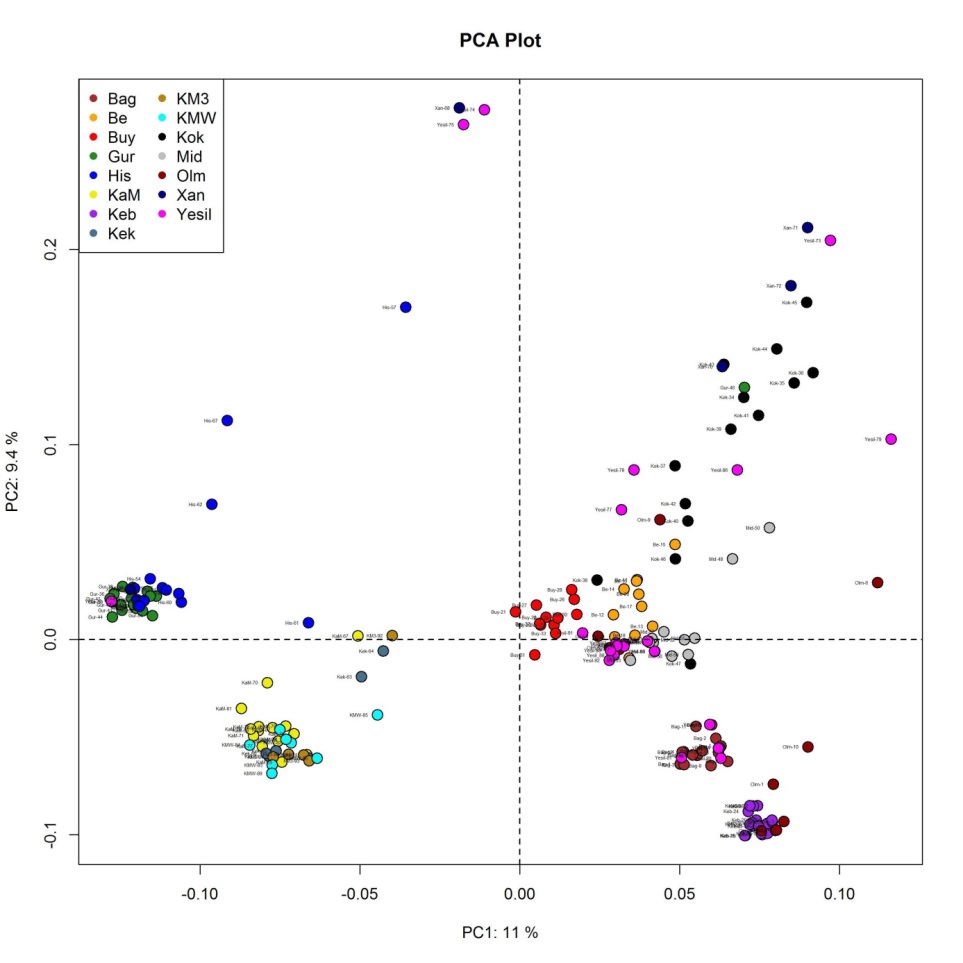

Supplement: S1 Fig — (DOCX) [file pone.0194056.s006.docx]

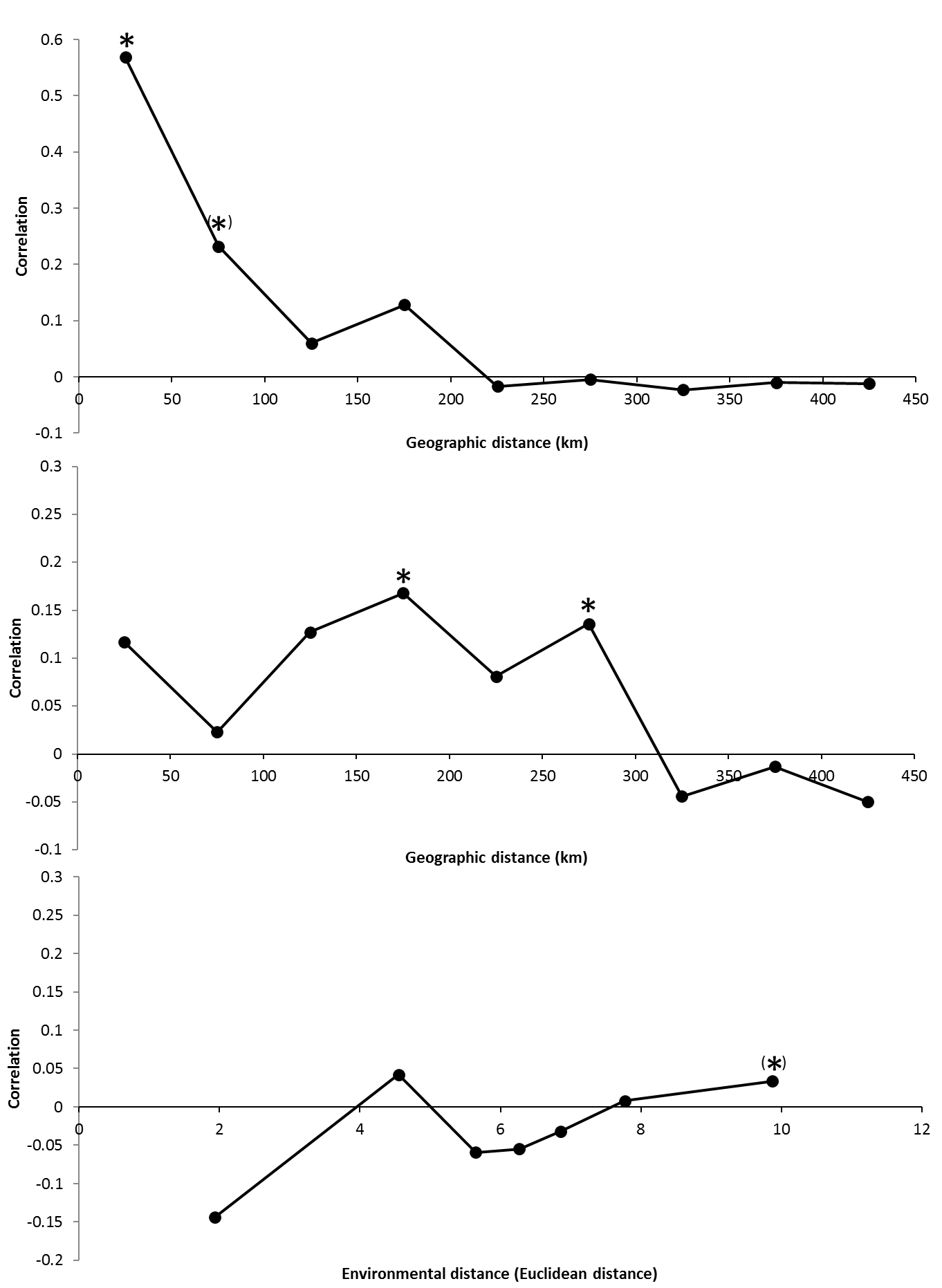

Supplement: S3 Fig — Positive correlation means higher environmental (a) or genetic (b, c) differentiation outside than inside the respective distance class. The significance of the normalized Mantel coefficient was calculated using a two-tailed Monte Carlo permutation test with 9999 permutations and the statistical significance of the coefficients was adjusted by Bonferroni correction. * P < 0.05, (*) 0.01 < P < 0.05 before significance correction. (DOCX) [file pone.0194056.s008.docx]

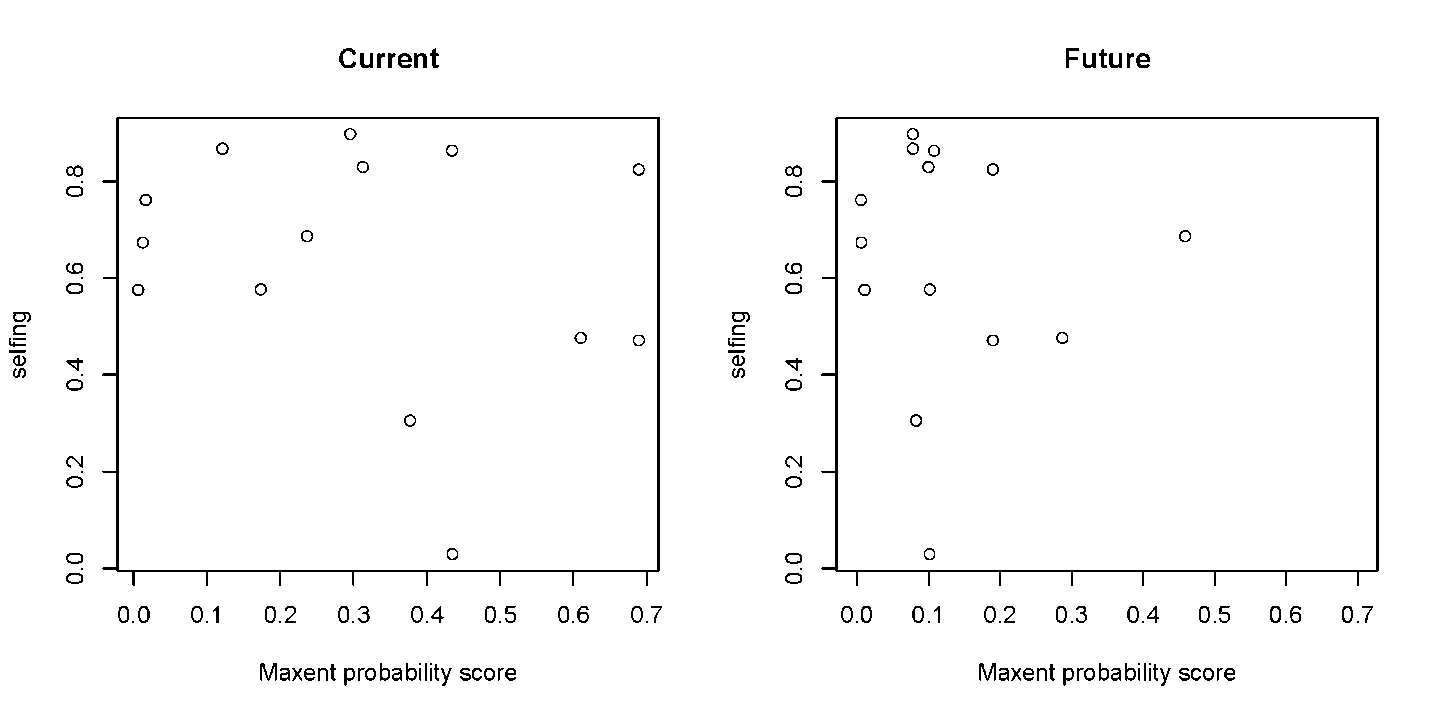

Supplement: S4 Fig — (DOCX) [file pone.0194056.s009.docx]
