# Supplementary material for: Genetic structure of wild pea (Pisum sativum subsp. elatius) populations in the northern part of the Fertile Crescent reflects moderate cross-pollination and strong effect of geographic but not environmental distance
Source: PLoS One. 2018 Mar 26;13(3):e0194056. doi: 10.1371/journal.pone.0194056 (PMC5868773; doi:10.1371/journal.pone.0194056)
Supplement: S2 Fig — Black points show mean distance of the distance groups. (DOCX) [file pone.0194056.s007.docx]

-0.2

-0.1

0

0.1

0.2

0.3

0.4

0.5

0

50

100

150

200

250

300

350

400

mean of interindividual distance groups [km]

Spatial autocorrelation analysis

kinship coefficient (Ritland 1996)
